# COVID-19 Infection and Circulating Microparticles—Reviewing Evidence as Microthrombogenic Risk Factor for Cerebral Small Vessel Disease

**DOI:** 10.1007/s12035-021-02457-z

**Published:** 2021-06-26

**Authors:** Che Mohd Nasril Che Mohd Nassir, Sabarisah Hashim, Kah Keng Wong, Sanihah Abdul Halim, Nur Suhaila Idris, Nanthini Jayabalan, Dazhi Guo, Muzaimi Mustapha

**Affiliations:** 1 Department of Neurosciences, School of Medical Sciences, Universiti Sains Malaysia, Health Campus, 16150 Kubang Kerian, Kelantan Malaysia; 2 Hospital Universiti Sains Malaysia, 16150 Kubang Kerian, Kelantan Malaysia; 3 Department of Immunology, School of Medical Sciences, Universiti Sains Malaysia, Health Campus, 16150 Kubang Kerian, Kelantan Malaysia; 4 Department of Internal Medicine, School of Medical Sciences, Universiti Sains Malaysia, Health Campus, 16150 Kubang Kerian, Kelantan Malaysia; 5 Department of Family Medicine, School of Medical Sciences, Universiti Sains Malaysia, Health Campus, 16150 Kubang Kerian, Kelantan Malaysia; 6 Translational Neuroscience Lab, UQ Centre for Clinical Research, the University of Queensland, Herston, Brisbane, 4029 Australia; 7 Department of Hyperbaric Oxygen, The Sixth Medical Center of PLA General Hospital, 6 Fucheng Rd, Beijing, 100048 China

**Keywords:** COVID-19, Coagulopathy, Microparticles, Cerebral small vessel disease, Stroke

## Abstract

Severe acute respiratory syndrome corona virus-2 (SARS-CoV-2) due to novel coronavirus disease 2019 (COVID-19) has affected the global society in numerous unprecedented ways, with considerable morbidity and mortality. Both direct and indirect consequences from COVID-19 infection are recognized to give rise to cardio- and cerebrovascular complications. Despite current limited knowledge on COVID-19 pathogenesis, inflammation, endothelial dysfunction, and coagulopathy appear to play critical roles in COVID-19-associated cerebrovascular disease (CVD). One of the major subtypes of CVD is cerebral small vessel disease (CSVD) which represents a spectrum of pathological processes of various etiologies affecting the brain microcirculation that can trigger subsequent neuroinflammation and neurodegeneration. Prevalent with aging, CSVD is a recognized risk factor for stroke, vascular dementia, and Alzheimer’s disease. In the background of COVID-19 infection, the heightened cellular activations from inflammations and oxidative stress may result in elevated levels of microthrombogenic extracellular-derived circulating microparticles (MPs). Consequently, MPs could act as pro-coagulant risk factor that may serve as microthrombi for the vulnerable microcirculation in the brain leading to CSVD manifestations. This review aims to appraise the accumulating body of evidence on the plausible impact of COVID-19 infection on the formation of microthrombogenic MPs that could lead to microthrombosis in CSVD manifestations, including occult CSVD which may last well beyond the pandemic era.

## Introduction

In 2020, the world is battling a pandemic caused by a novel coronavirus disease 2019 (COVID-19). The first appeared in Wuhan, China, in December 2019 with 41 cases of atypical pneumonia; it was not until early January 2020 that these cases were confirmed as an infection attributed to COVID-19 [1]. The pneumonia it caused was later named as severe acute respiratory syndrome coronavirus-2 (SARS-CoV-2) [2]. By early March 2020, COVID-19 has been declared as a pandemic by the World Health Organization (WHO), and to date, it remains unabated worldwide surpassing 100 million cases and over two million deaths as on 15 January 2021 [3]. The clinical manifestations of COVID-19 and the disease course are erratic, ranging from asymptomatic to mild respiratory infections, pneumonia to acute respiratory distress syndrome (ARDS), and even death [4, 5]. At present, we have no definitive treatment for COVID-19, while concerted global efforts are well in progress [6]. Given that our present knowledge of COVID-19 and SARS-CoV-2 is still expanding, most countries are currently putting their best efforts by implementing preventive and control strategies to break the chain of COVID-19 infection.

Beyond the pulmonary manifestations, both direct and indirect consequences from COVID-19 infection are known to cause cardio- and cerebrovascular complications [7]. Despite current limited knowledge on COVID-19 pathogenesis, inflammation, endothelial dysfunction, and coagulopathy appear to play critical roles in COVID-19-associated acute cerebrovascular disease (CVD) [8, 9]. One of the major subtypes of CVD is cerebral small vessel disease (CSVD) which represents a spectrum of pathological processes of various etiologies affecting the brain microcirculation that can trigger subsequent neuroinflammation and neurodegeneration. Prevalent with aging, CSVD is a recognized risk factor for stroke, vascular dementia, and Alzheimer’s disease (AD) [10, 11]. In the background of COVID-19 infection, the known heightened cellular activation from inflammation and oxidative stress may result in elevated levels of microthrombogenic extracellular-derived circulating microparticles (MPs). Consequently, MPs could act as pro-coagulant risk factor that could serve as microthrombi for the vulnerable microcirculation in the brain leading to recognized CSVD manifestations [12, 13], i.e., from asymptomatic (occult) to symptomatic (typical lacunar stroke).

Hence, this review aims to appraise the accumulating body of evidence on the plausible impacts of COVID-19 on the formation of microthrombogenic MPs that could lead to microthrombosis in CSVD manifestations, including occult CSVD which may last well beyond the pandemic era.

## Characteristic of COVID-19

The family *Coronaviridae* are large, enveloped viruses with a positive sense ribonucleic acid (RNA) genome that can infect both animals and humans. These coronaviruses may resemble one another in terms of their pathogenesis and pathological features and even share similar clinical manifestations [14]. Bats are widely viewed as its reservoir, while Malayan pangolins (*Manis javanica*) is thought to be the intermediate host to facilitate the zoonotic transfer to humans [15].

According to the International Committee on Taxonomy of Viruses, SARS-CoV-2 belongs to a member of the genus *Betacoronavirus* [16] that can cause multi-system clinical manifestations involving respiratory, enteric, hepatobiliary, and nervous systems [17]. SARS-CoV-2 has now proven itself as a highly pathogenic coronavirus to infect human populations. Two other members of this family, the severe acute respiratory syndrome coronavirus (SARS‐CoV) and Middle East respiratory syndrome coronavirus (MERS‐CoV), had previously resulted in significant global outbreaks in 2002 and 2012, respectively [18], though not to the scale of a pandemic. SARS-CoV-2 is genetically distinct from SARS-CoV (near 79% similarity) and MERS-CoV (near 50% similarity) [1]. Structurally, SARS-CoV-2 RNA encodes four principal proteins: one nucleocapsid protein surrounding the RNA genome and three membrane proteins, the spike glycoprotein (S) with S1 and S2 domains, the matrix glycoprotein, and the envelope protein [19].

### The Virology of COVID-19

During the initial phase of the infection, the virus infiltrates and proliferates in the lung parenchyma. Upon entry into the respiratory tract, the virus targets the surfactant-producing, alveolar epithelial type 2 (AT2) cells. Surfactant decreases the surface tension within alveoli to reduce airway collapse. This early phase is characterized clinically by mild constitutional symptoms as the virus releases inflammatory mediators to stimulate monocyte/macrophage infiltration as the innate immune system initial response [20].

**Fig. 1:**
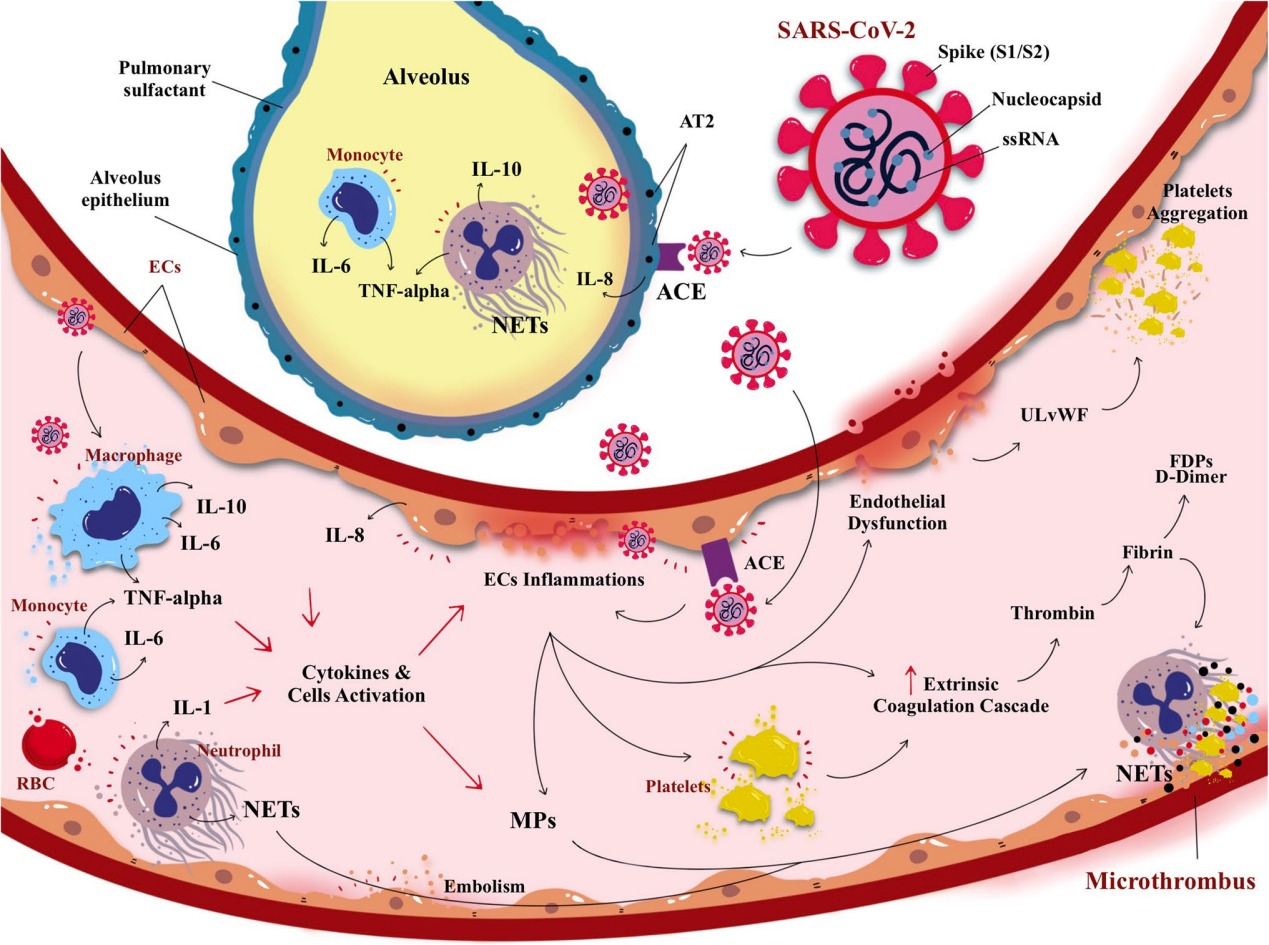
A schematic illustration of direct SARS-CoV-2 infection from the lung alveolus and blood circulation. The virus SARS-CoV-2 (and its main structure) acquired through respiratory droplets attacking angiotensin converting enzymes (ACE) type 2 receptors that are present on the surface of alveolus epithelium, namely, the alveolar epithelial type (AT2). The attachment of SARS-CoV-2 with ACE elicited the inflammatory reaction of the AT2 cells, releasing pro-inflammatory cytokines; i.e., interleukin-8 (IL-8) alongside the activation of monocytes and neutrophil elevate the inflammation causing lung parenchymal injury. In addition, SARS-CoV-2 also can enter blood circulation and raise activation of circulating cells (i.e., macrophage, monocytes, platelets, and neutrophil) to release pro-inflammatory cytokines causing endothelial inflammation (endotheliitis). Endotheliitis then activates the coagulation cascade and production of thrombin followed by fibrinolysis and fibrin. If left untreated, the infection will progress to cause hypercoagulation state leading to coagulopathy. Collectively, cytokines, cellular activation, and endothelial inflammation drive the production of microparticles (MPs) which further instigate the production of microthrombus, cell-endothelium adhesion, and aggregation0

The entry into the AT2 cell is mediated by S glycoprotein interaction with the host angiotensin converting enzyme 2 (ACE2) receptor [21] (Fig. 1). Of note, ACE2 receptors can also be found in the kidney, heart, gut, pancreas, and endothelial cells (ECs) [22]. In normal physiology, ACE2 helps to regulate the blood pressure via inhibition of the angiotensin renin-aldosterone pathways [23]. However, elevated level of angiotensin II has been associated with vasoconstriction oxidative process and apoptosis that lead to neurodegeneration and age-related degenerative disease [24]. The S1 domain facilitates the virus-receptor binding, while the S2 domain causes fusion of the viral RNA with the cell membrane [24]. Notably, the CoV S protein is cleaved by a group of serine proteases, including elastase, cathepsins, trypsin, type 2 transmembrane serine protease (TMPRSS2) [20], and integrins that enable invasion into the epithelial cells [25]. On this basis, the use of chloroquine and hydroxychloroquine is linked to their ability to increase endosomal pH which can prevent ACE2 separation from SARS-CoV-2 [26] and, hence, guard against an intracellular virus diffusion. The anti-viral drugs (remdesivir, ribavirin, favipiravir, umifenovir, lopinavir/ritonavir) interfere with RNA processing steps to arrest the viral replication [27]. Furthermore, neutralizing antibodies from those who recovered from COVID-19 had resulted in reduction of the viral loads [28]. Meanwhile, candidate vaccines with promising leads include adenovirus recombinant vectors, type 26 (rAd26) and type 5 (rAd5) carrying the gene for SARS-CoV-2 spike glycoprotein (rAd26-S and rAd5-S) and the chimpanzee adenovirus-vectored vaccine (ChAdOx1 nCoV-19) (Oxford University/AstraZeneca) expressing the SARS-CoV-2 spike protein [29, 30]. Moreover, two encapsulated RNA-based vaccines have been proven effective including mRNA-1273 (Moderna) with 94.1% efficacy [31] and BNT162b2 (Pfizer/BioNTech) with 95% efficacy [32] with emergence use approvals by regulatory bodies in over 70 countries worldwide to date and counting. Aside from vaccines, there are also efforts to synthesize recombinant immunoglobulins to mimic the endogenous, neutralizing antibodies [19]. Numerous clinical trials are also underway that target antiprotease activities, including the plasmin(ogen) inhibitor, tranexamic acid, or TMPRSS2 antagonist (camostat mesylate and nafomastat) [33].

### Clinico-pathological Features of COVID-19

COVID-19 affects all ages with adult predominance [4]. The established risk factors include age greater than 65 years, diabetes mellitus (i.e., type 2 diabetes mellitus, T2DM) and hypertension in nearly 40% of cases [7]. The advocated physical distancing is a measure to minimize human-to-human transmission that can occur through droplets from the infected respiratory tract which can reach up to 2 m from a sneeze or a cough of an infected person [34]. The average incubation period is between 1 and 14 days [33], with up to 25% of those tested positives for COVID-19 that are asymptomatic [5, 35]. Upon entry across the mucous membranes (nose and/or larynx), COVID-19 viruses can track its way to reach the lung parenchyma and subsequently result in viremia once in the systemic circulatory system resulting in a widespread hyperinflammation (Fig. 1). The commonest symptoms include fever, dry cough, dyspnea, ageusia, anosmia, myalgias, and/or fatigue [36]. Majority of cases would also have radiological evidence pneumonic changes [37]. Hence, the infected persons could worsen clinically, between 7 and 14 days after the onset [38].

**Fig. 2:**
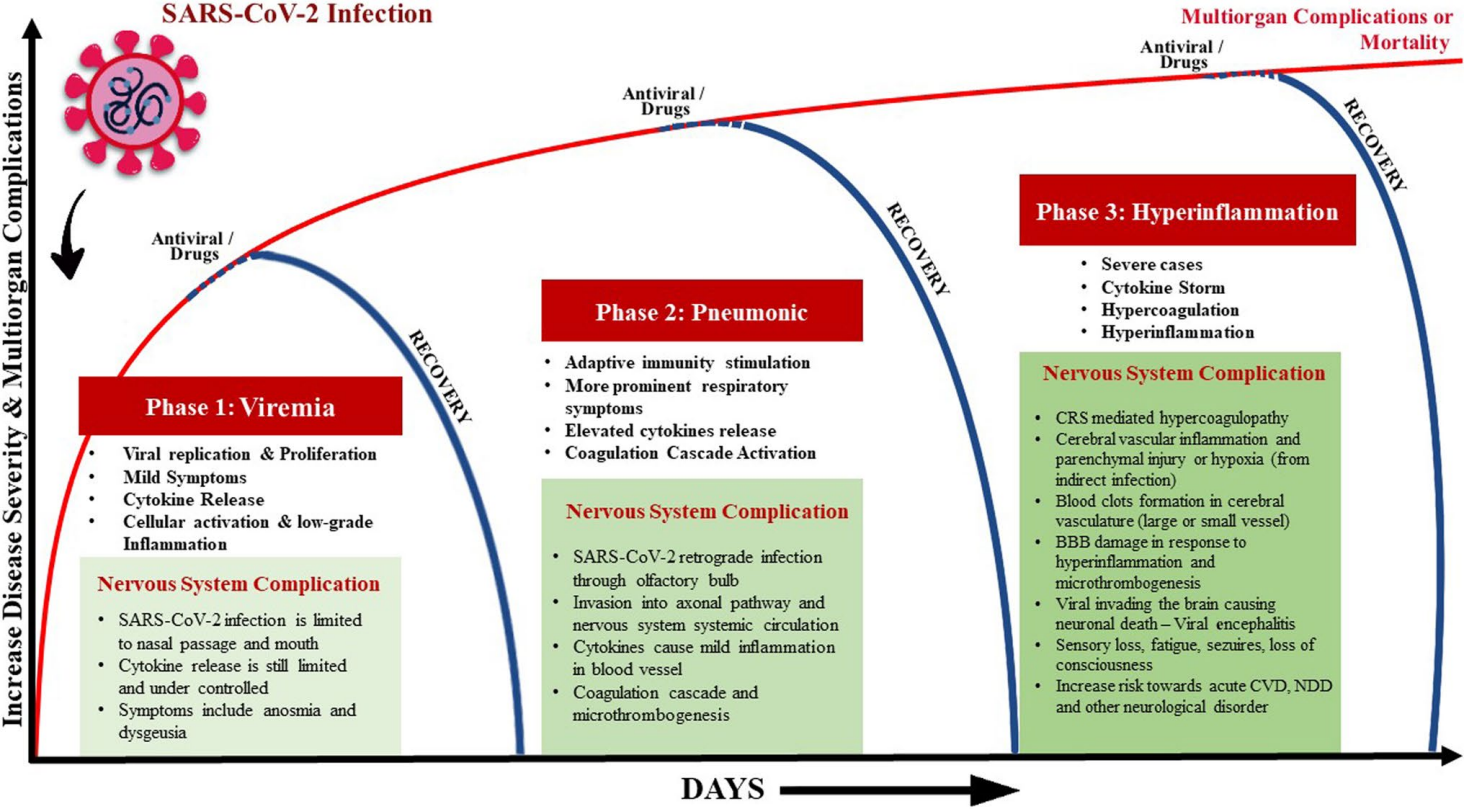
The known phases of COVID-19 infection: from viremia, pulmonary to multi-system manifestations; emphasis made on the impacts on the nervous system. Also shown are the simplified underlying COVID-19 likely pathomechanisms as the infection progresses, and the current corresponding therapeutic targets in the clinical management of each phase. It is probable that even after the recovery at any phase of the disease, the involvement of cellular activation by-product (such as microparticles) may persist and result in an undesirable health sequel

Based on the current targeted therapies for COVID-19 cases that correspond to some extent with the evolving pathophysiological processes, the infection has been proposed to constitute three key phases [39, 40]: The first phase involves viral replication and manifests with mild symptoms (viremia phase); the second phase features adaptive immunity stimulation and more prominent respiratory symptoms (pneumonic phase); and the last phase, in severe cases, is characterized by a hyperinflammation phase (Fig. 2). However, overlaps do exist between these phases in individual patients. In general, individuals with competent immune functions and without notable risk factors may effectively suppress the virus in the first and/or second phase. However, patients with immune-compromised conditions may have a higher risk of progressing to the severe hyperinflammation phase leading to death.

Moreover, the lung parenchymal injury is mediated by inflammatory responses with vasodilation, endothelial permeability, and leukocyte recruitment (Fig. 1). The features of respiratory dysfunction in this phase are distinct from the typical ARDS. The pulmonary compliance is slightly decreased in intubated COVID-19 patients [41] and responsive to prone positioning and moderate levels of positive end expiratory pressure oxygen therapy. Unlike typical ARDS where alveoli are primarily affected [42], combination of severe hypoxemia without significant reduction in pulmonary compliance is rare. However, in COVID-19, it disrupts the pulmonary vascular endothelium resulting in a diffuse systemic disease. This is aggravated by a rapid activation of the coagulation cascade, which leads to pervasive micro- and macro-thrombogenesis within the lungs and other organs. Evidently, a markedly raised D-dimer level has been associated with a worse prognosis. Pulmonary vascular occlusion caused by the thrombotic microangiopathy and/or pulmonary embolism leads to accumulation of respiratory dead space. Hence, this inflicts further damage to the lung whereby some patients could progress into ARDS-like features [41, 42]. Thus, multiple pathomechanisms are being implicated in the progression to ARDS in COVID-19 that feature disproportionate endothelial damage with hypoxic pulmonary vasoconstriction (i.e., ventilation-perfusion mismatch), hypoxemia, as well as thrombogenesis [43]. In some cases, these occur within the background of heightened inflammatory responses that progress into the next phase of the infection.

### COVID-19 Complications

It is recognized that even with reducing viral loads, some COVID-19 patients continue to mount heightened inflammatory storms leading into the final, hyperinflammation phase of the disease. This phase features systemic inflammation and distant organ damage that cause multi-organ dysfunction syndrome (MODS) [39, 44] (Fig. 2). Several serum markers found to be elevated and may influence prognosis include C-reactive protein (CRP), pro-inflammatory cytokines such as interleukin (IL)-2, IL-6, IL-7, interferon-γ inducible protein 10 (IP-10), granulocyte-colony stimulating factor (G-CSF), tumor necrosis factor alpha (TNF-α), macrophage inflammatory protein 1 alpha (MIP-1α), and monocyte chemoattractant protein 1 (MCP-1) [45–48].

Hence, these pathophysiological mechanisms lead to both focal and systemic microvascular inflammation, which in turn trigger endothelial activation and aggravate the prothrombotic states (Fig. 2). The massively elevated serum D-dimer levels may also be due to the vascular disease reported in this phase. Clinically, a significant number of hospitalized COVID-19 patients also suffered from acute pulmonary vascular thrombosis or embolism, myocardial infarction, CVD, and systemic arterial thrombosis that worsened their prognosis [7]. There are amplified fibrin degradation products (FDPs) reported in most severe cases with ARDS, septic shock, concurrent bacterial infections, disseminated intravascular coagulopathy (DIC), and MODS [45, 49]. In such cases, heparin has been used as part of the multi-therapy regime. Thus, given this immune-coagulation systems interaction, heparin inhibition of thrombin activity may attenuate the inflammatory storms [50]. Similarly, potentials of corticosteroids, tocilizumab, sarilumab, and monoclonal antibodies against IL-6 receptor are actively being pursued to mitigate the severity of this phase.

## COVID-19 and Cerebrovascular Disease

It is well known that viral infections can inflict severe damage to the structure and function of the nervous system, for example, viral infection in central nervous system (CNS) causing encephalitis and acute demyelinating lesions while systemic viral infections causing toxic encephalopathy [51]. Numerous evidence and case series reported SARS-CoV-2 complications are not limited to respiratory system but include obvious manifestations of neurological disturbances including seizures, anosmia, stroke, encephalopathy, confusion, acute CVD, and total paralysis [9, 52–54]. It is estimated about 20% of COVID-19 patients admitted to the intensive care unit (ICU) exhibited neurological consequences with a higher risk of mortality [55, 56]. Given the wide-ranging healthcare accessibility to COVID-19 patients management worldwide, these neurological symptoms may manifest until after discharge from COVID-19 hospitalization, and some may even result in death [57]. Alarmingly, recovered COVID-19 patients are believed to be at a possible higher risk for long-term effects of neurodegenerative, neurocognitive, and neuropsychiatric disorders such as dementia, depression, anxiety, AD, and Parkinson’s disease [58, 59] (Fig. 2).

Furthermore, gradual reports have emerged since the outbreak of COVID-19 demonstrating the link between COVID-19 and CVD/acute CVD among those with a higher risk of cardio-embolic as well as arterio-arterial embolic events [54, 57, 60, 61]. Through neuroimaging, Li et al. [62] confirmed the evidence of CVD and reported that most COVID-19 patients had cerebral ischemic infarcts in both large and small arterial vessels [62]. This is in parallel with the observed complication of DIC which is higher among COVID-19 patients with higher FDPs levels and prolonged prothrombin time (PT) and activated partial thromboplastin time (aPTT) [57, 62], with risk of death. In Wuhan, where the disease originated, about 36% of patients with COVID-19 showed signs and symptoms of acute CVD, where up to 6% among those with a more severe disease (elevated D-dimer and depleted platelets) [9, 63, 64]. The common types of acute CVD associated with COVID-19 infection in most reports are cryptogenic strokes (65%) [60]. Nevertheless, the causality between CVD and COVID-19 infection remains obscure, although recognized multi-factorial triggers include hypercoagulability, hyperviscosity, thrombogenesis, and cytokines release syndrome (CRS) as observed in growing case series [46, 57, 63] (Fig. 1).

### Cerebral Small Vessel Disease

Accumulating body of evidence implicates SARS-CoV-2 role in eliciting the systemic event (i.e., inflammation and pro-coagulant/thrombotic cascade) within the large vessel environment linking COVID-19 with large vessel strokes. Hence, similar repercussion may well extend into the small vessel microenvironment within the brain [65]. Since the involvement of small vessel disease (SVD) has been confirmed as a complication from COVID-19 infection, it is important to appreciate that one of the most significant manifestations of SVD (i.e., stroke) occurs from the occlusion (ischemia) of small blood vessels deep within the brain or so-called cerebral ischemia or ischemic stroke [66]. Prevalent among healthy aging adults, about 30% of ischemic or lacunar strokes are thought to represent CSVD [66]. CSVD is due to the spectrum of complex and overlapping pathophysiological mechanism and often occult or asymptomatic in nature that often incidentally found after neuroimaging (i.e., magnetic resonance imaging, MRI). However, it is well supported that CSVD is mainly due to the pathological consequences of SVD on the brain parenchyma rather than the underlying diseases of the vessels [67]. Therefore, the term CSVD signifies a brain parenchyma injury (often progressive or accumulating) associated with distal leptomeningeal and intracerebral vessel pathology that resides in poorly collateralized subcortical gray and deep white matter. Moreover, it is mainly due to several focal or diffuse microvascular pathological processes that affect and cause occlusion to the small perforating cerebral capillaries (of sizes 50–400 mm), small arteries (mostly branches of MCAs), arterioles (diameter < 0.1 mm), and venules that penetrate and supply the brain cortical and subcortical region [68, 69].

**Fig. 3:**
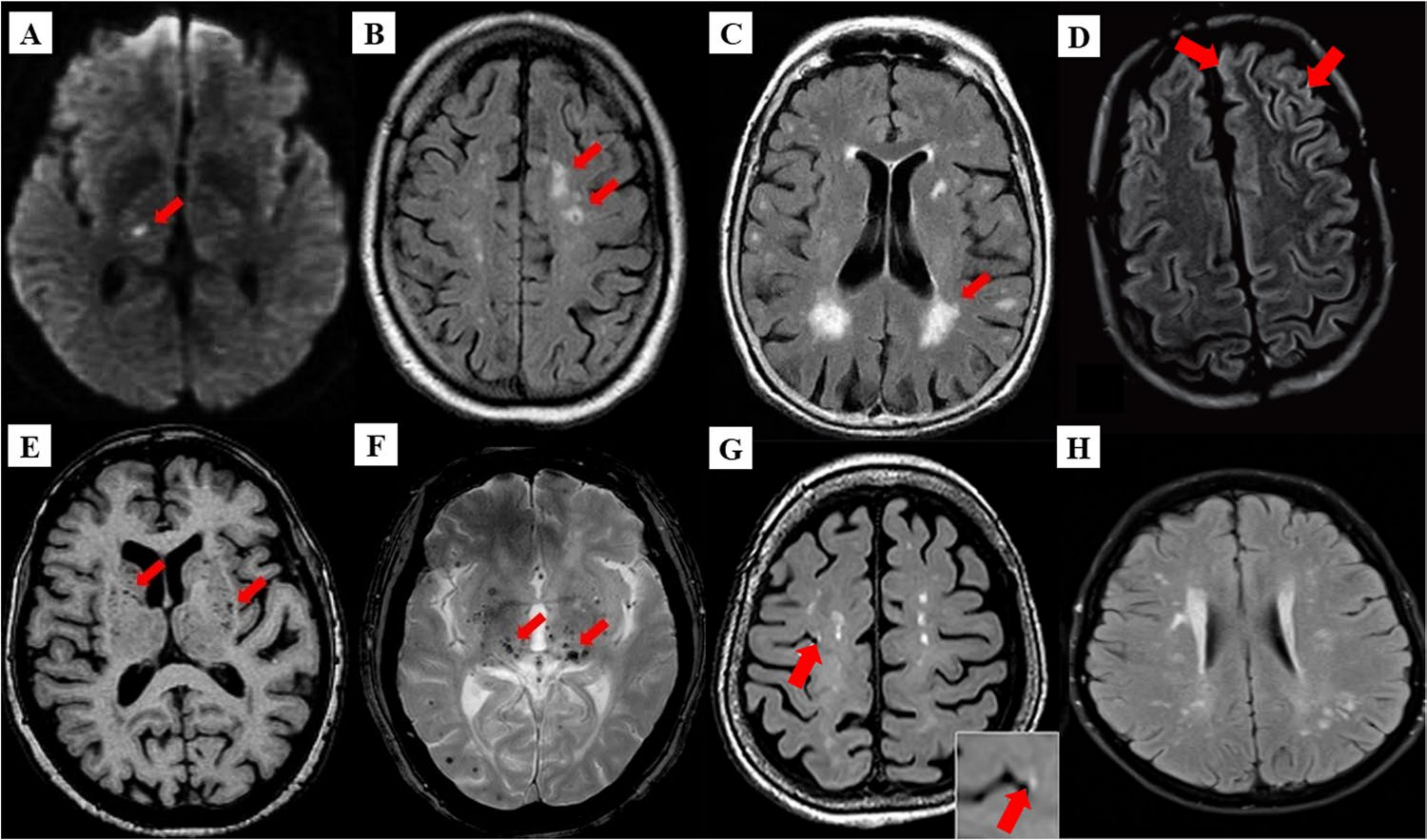
Neuroimaging correlates of CSVD based on STRIVE method. **A** Recent small subcortical infarct (RSBI) on diffusion weighted imaging (DWI) (red arrow). Usual diameter is around 3–15 mm, with hyperintense rim surrounding ovoid cavity. RSBI seen as increased T2-weighted, fluid attenuated inverse recovery (FLAIR), and DWI signal intensities and decreased T1-weighted signal and iso-intense in T2*-weighted gradient recoiled echo (GRE) signal and susceptibility weighted imaging (SWI). RSBI is best identified through DWI with usual infarct diameter of ≤ 20 mm. **B** Lacunar infracts on FLAIR (red arrow). Lacunar infarcts appeared as increase hyperintensity in T2-weighted signal, decrease T1-weighted, and FLAIR signal and iso-intense in DWI. Usual diameter is around 3–15 mm, with hyperintense rim surrounding ovoid cavity. **C** White matter hyperintensities (WMHs) of presumed vascular origin on FLAIR (arrow). WMHS seen as increase intensity or hyperintensity on T2-weighted imaging, T2*-weighted GRE and FLAIR (best identified); iso-intense on DWI; and hypointense (decrease intensity) on T1-weighted imaging. **D** FLAIR WMHs at left superior frontal gyrus and left anterior cingulate cortex, from a 60-year-old COVID-19 patient without history of seizures. **E** Enlarged perivascular spaces (PVS) on T1-weighted imaging (red arrow) with usual diameter of ≤ 2 mm. PVS is seen as decrease FLAIR and T1-weighted signal intensity, with increase T2-weighted signal. Meanwhile, T2*-weighted GRE and DWI appeared iso-intense, and they also appeared in similar signal intensity with cerebrospinal fluid (CSF). **F** Cerebral microbleeds (CMBs) on T2*-GRE (red arrow). CMBs are small, rounded areas of signal void with blooming, whereby they were visualized as iso-intense T1- and T2-weighted signal, FLAIR, and DWI. They are best identified under T2*-weighted GRE or SWI as reduced signal intensities. Usual diameter is around ≤ 10 mm (mostly 2–5 mm). **G** 3 Tesla-MRI representation of cortical microinfarcts (red arrow) on T1-weighted (hypointense). **H** FLAIR WMHs in multiple foci, including the deep white matter, periventricular, and subcortical regions in COVID-19 patient with CADASIL. Notes: (**A**), (**B**), (**C**), (**E**), and (**F**) were adapted from Mustapha et al. [70]; (**D**) was adapted from Muhammedi et al. [73]; (**G**) was adapted from Takasugi et al. [74], and (**H**) was adapted from Williams et al. [75]

There are several etiopathogenic classifications of CSVD. However, the most well-recognized forms of CSVD are the amyloidal CSVD (e.g., sporadic, and hereditary cerebral amyloid angiopathy [CAA]) and non-amyloidal CSVD including age-related and vascular risk factor-related SVD (i.e., arteriolosclerosis and age) [68]. Other less common forms of CSVD include inherited or genetic (monogenic) CSVD that is recognizably different from CAA (i.e., Fabry’s disease and cerebral autosomal dominant arteriopathy with subcortical ischemic strokes and leukoencephalopathy [CADASIL]), inflammatory and immunologically mediated CSVD, venous collagenosis, and other CSVD (i.e., non-amyloid micro-vessel degeneration in AD and post-radiation angiopathy) [70]. Several manifestations of CSVD can be seen through clinical, such as acute lacunar infarct and intraparenchymal hemorrhage, and radiological (i.e., neuroimaging), such as white matter hyperintensities (WMHs) of presumed vascular origin, cerebral microbleeds (CMBs), cortical microinfarcts, lacunar infarcts and recent subcortical brain infarcts (RSBI) and enlarged perivascular spaces (PVS), or pathological phenomena with multifaceted etiologies [13, 69, 71]. However, the lack of standardization and consistency in neuroimaging techniques lead to the development of STandards for Reporting Vascular changes on nEuroimaging (STRIVE), aided in the imaging based visual identification and classification of CSVD spectrum [72]. Figure 3 describes the neuroimaging correlates of different CSVD manifestations based on the STRIVE method and COVID-19 findings.

### COVID-19 and CSVD: Inter-current Risk Factors

A recent study has highlighted that young and healthy individuals either symptomatic (i.e., cough or fever) or asymptomatic of COVID-19 can present with large vessel stroke [76], while hypertensive COVID-19 patients are more likely to develop CVD, both large and small vessel strokes [77]. Moreover, metabolic syndromes such as obesity and high body mass index (BMI) have been associated with the severity of respiratory viral infection [78] and COVID-19, hence being considered a risk factor for acquiring the infection [79, 80]. However, there are several and complex known risk factors towards development and progression of CSVD manifestation. For example, increase in WMHs, lacunar infarcts, and recent subcortical brain infarct (RSBI) were associated with lifetime exposure towards cardio-cerebrovascular risks such as metabolic syndrome (i.e., hypertension, obesity, hyperlipidemia, dyslipidemia), lifestyle (i.e., smoking, extreme alcohol intake), and T2DM which can progress towards acute ischemic (lacunar) stroke [81]. Apparently, age has served as one of the most significant determinants of the onset, proportion, and progression of all CSVD manifestation (prevalent with healthy aging [~ 6%] in CMBs). A higher risk of CMBs has been found in individuals with symptomatic CVD such as ischemic stroke and intraparenchymal hemorrhage [11]. Therefore, metabolic syndromes and age are major risk factors for CVD-related COVID-19 infection.

Meanwhile, genetic factors such as *NOTCH3* gene (chromosome 19) mutation as seen in CADASIL; mitochondria DNA mutation as seen in mitochondrial encephalomyopathy; lactic acidosis; and stroke like syndrome (MELAS), Fabry’s disease, and familial CAA increase the burden and prevalence of CSVD [82]. One case report demonstrated the presence of chronic SVD in a young (38 years old) COVID-19 patient with a family history of CADASIL, whereby bilateral acute cerebral infarcts in multiple locations within internal border zone (or subcortical lesion) distribution (i.e., at the junction of two arterial territories) were found after neuroimaging. Hence, this suggests the involvement of small vessel attributable to compromised cerebral microcirculation [75] (Fig. 3).

Intriguingly, a recent retrospective case–control study (41 COVID-19 cases) had shown that COVID-19 was an independent risk factor for cerebral ischemia (i.e., acute ischemic stroke), whereby the association was achieved even after age, sex, and other risk factors (i.e., hypertension, T2DM) were adjusted and matched [83], suggesting that age is not associated with COVID-19-mediated CVD. This is supported by Moriguchi and colleagues [84] who reported hyperintense signal on brain MRI in the hippocampus and inferior horn of the right ventricle and right mesial temporal lobe in a young (24-year-old) COVID-19 patient [84] that indicated the presence of CSVD manifestation. Additionally, a case report highlighted the involvement of cerebral microcirculation insults with a COVID-19 test positive but asymptomatic young individual (i.e. without COVID-19 flu-like symptoms) who suffered from a sudden onset dysphasia and left hemiparesis where subsequent neuroimaging revealed two recent small infarctions in the right perirolandic cortex (hence, involving small vessel) without signs of any previous ischemic/hemorrhagic lesion and with rigorous exclusions of other conventional stroke risk factors [85]. Thus, the involvement of symptomatic small vessel stroke is recognized in COVID-19 small case series and often regarded as cryptogenic [57, 63].

Additionally, Hanafi and colleagues [86] had reported the possibility of a small intracranial vascular injury in the distribution of cerebral distal perforating arteries (i.e., enlarged PVS, deep WMHs) without large vessel (intra- or extracranial) involvement as COVID-19 neurovascular complications in an older individual suggestive of small vessel damage [86], while Brun and colleagues [87] reported the involvement of acute demyelination (restricted diffusion with FLAIR-MRI hyperintensities) as seen from bilateral and asymmetrical periventricular (involving corpus callosum) and deep WMHs of a 54-year-old COVID-19 patient, hence suggesting cerebral ischemia due to small vessel vasculitis [87]. Meanwhile, a prospective study conducted on 60 recovered COVID-19 patients (age- and sex-matched) found more than half (55%) presented with neurological syndromes [88]. Neuroimaging study (including diffusion tensor imaging, DTI) revealed that these individuals had bilateral enlargement of gray matter volumes (GMV) in their central olfactory system, suggesting that SARS-CoV-2 may invade CNS through olfactory bulb via retrograde route and the GMV enlargement indicates neuronal compensation during recovery period after COVID-19 [88]. Besides, DTI parameters revealed that these individuals had higher mean diffusivity (MD) and lower fractional anisotropy (FA) in white matter tracts (i.e., corona radiata, external capsule, and superior fronto-occipital fasciculus) [88], suggesting an increase in white matter fibers alignment and limited diffusion prior to an intrinsic neuronal remyelination after an infection or during the recovery period [89]. Collectively, there is enough evidence to deduce the likely cerebral microcirculation (structural and function) disruption and cerebral white matter loss of integrity during and after (i.e., recovery period) COVID-19 infection, hence indicating the risk and consequences of COVID-19 on onset and progression of CSVD even after the infection has ceased, be it asymptomatic or symptomatic manifestations.

## COVID-19 and CSVD: Putative Pathomechanisms

Relatively small micro-vessels play essential roles in CNS in terms of neurovascular unit or the blood–brain barrier (BBB). To date, various and intensive investigations have been carried out to study the mechanism of interaction between cerebral parenchyma and its surrounding microvasculature [90]. However, it is well accepted that neurovascular unit or BBB owns the prior role in brain health and plasticity (capacity to recover) from insults that may initiate the pathologic cascade towards neurodegenerative disease (NDD). Two classical clinicopathologic representations of CSVD are linked to arteriolosclerosis or lipohyalinosis (thickening and/or damage the wall of arterioles) and occlusion of cerebral penetrating arteries [91]. However, most of the SVD are representation of cerebral arterial microcirculation flow obstruction (intrinsic or extrinsic). For example, an arteriolar occlusion or narrowing resulted in ischemia as seen in small lacunar infarcts.

Various pathological changes of CSVD not only give rise to cerebral parenchyma damage (i.e., axonal injury, neuronal apoptosis, demyelination, and oligodendrocyte damage), with consequent neurological symptoms, signs, and multifaceted neuroimaging findings [92]. Nonetheless, the underlying pathomechanism of CSVD remains contentious despite the growing insights from histopathological, epidemiological, and physiological studies. Several systemic dysregulations including abnormal coagulation, elevated microthrombosis, genetic mutation, increase cellular activation, inflammation, and oxidative stress are the major contributors towards endothelial dysfunction, altered cerebral blood flow (CBF), and BBB breakdown which provide further insights on the current known pathomechanism of CSVD.

Moreover, during the course of COVID-19 infection, the hypercoagulability and thrombotic vascular events are known to be associated with neurovascular involvement such as acute CVD [52]. Interestingly, SARS-CoV-2 has been detected in the cerebrospinal fluid (CSF) indicating its direct ability to invade and infect the nervous system from ACE2 receptor-mediated entry through retrograde route [52]. Hence, various studies have proposed several plausible mechanisms of COVID-19-related nervous system damage including direct infection injury such as viral neurotropism through neuronal pathway (retrograde route) and systemic blood circulation or hematogenous route (i.e., endothelial dysfunction, coagulopathy, inflammation). Other indirect infection also has been proposed such as cardio-embolism and viral proliferation in the lung that mediate hypoxia injury and immune injury [52, 93]. These proposed mechanisms are associated with the current spectrum of dynamic cerebral microvascular pathological process towards the onset and progression of CSVD. Hence, the foregoing sections will deliberate on the potential pathomechanism of COVID-19-related CSVD.

### COVID-19 Neurotropism: Neuronal Pathway

Researchers have reported the presence of viral genetic materials and proteins from the samples of nervous tissues such as CSF or brain tissues, suggesting neurotrophic properties of viruses whereby they can directly invade the nervous tissues that trigger subsequent immune responses from nerve cells such as microglia, macrophages, or astrocytes and cause nervous system injury [94–96]. After the invasion into the neural tissues, the viruses can migrate and further infect the sensory or motor neurons, hence achieving anterograde or retrograde transport aided by motor proteins (i.e., kinesins and/or dynein) [97]. In this case, olfactory neuronal transport is the main example. Evidently, in COVID-19 early viremia phase, many patients have reported anosmia and dysgeusia, probably from the spread through the olfactory epithelium or cribriform bone in the nasal cavity and reach the brain through retrograde route transfer, thought to occur within seven days after infection in the respiratory tract [98]. A recent study had confirmed through genomic sequencing that new pneumonic virus such as SARS-CoV-2 were present in CSF, neural, and cerebral capillary ECs of COVID-19 patients, supporting the fact that SARS-CoV-2 can infect the CNS from peripheral neuronal pathway and mediate further nervous system damage [99, 100]. Interestingly, one pre-clinical animal study had shown that the removal of the olfactory bulb from mice inhibited the direct invasion of SARS-CoV infection into the CNS [101].

Furthermore, in the pneumonic phase of the infection, the presence of ACE2 receptors in glial cells in the brain and neurons in the spinal cord provide access to the virus proliferation [97]. In this case, SARS-CoV-2 spike protein interaction with ACE2 receptors invades the capillary endothelium, hence breaching the BBB to infiltrate the nervous system [61, 63], and increases the risk towards CSVD and other neurovascular disease. The involvement of the renin-angiotensin system (RAS) also leads to an exaggerated blood pressure increment that poses a risk of acute cerebral hemorrhage [63, 64]. In the final, hyperinflammatory phase, the presence of CRS with further deterioration in the neurological status of COVID-19 patients may result in altered sensorium, seizures, or even death [64]. Furthermore, neurotropic nature of the virus could also activate glial cells and induce a pro-inflammatory state that correspond with the elevated serum levels of inflammatory markers such as IL-6, IL-12, IL-15, and TNF-α [64].

Apart from direct infection through neuronal pathway, another recent report had shown SARS-CoV-2 direct infiltration through hematogenous route of cerebral small vessel-ECs, causing endothelial inflammation and dysfunction or endotheliitis [87]. Subsequently, such endotheliitis would lead to cerebral vasoconstriction, BBB damage, and cerebral vasculitis and likely to pose imminent cerebral ischemic damage (given the proximity to deep and periventricular white matter) despite an apparent absence of overt neurological symptoms [43, 87]. Thus, little is known at present on the likely impact of such COVID-19-related disease mechanisms or complications on the brain small vessel microenvironment, especially on the recognized asymptomatic manifestation of CVSD. Therefore, the foregoing sections will elaborate on the COVID-19-mediated direct complications that may contribute to the pathomechanism of CSVD through several processes including cytokine storm-mediated hyperinflammation, oxidative stress, coagulopathy, cellular activation, and microthrombosis.

### Cytokine Storm and Oxidative Stress

SARS-CoV-2 infection in COVID-19 patients has been widely reported to induce cytokine storm or CRS, whereby a burst of cytokines release triggers the hyperinflammation and immune cells infiltration in their lungs [102, 103] (Fig. 1). Such a phenomenon may also be the factors of onset for acute CVD [46, 49]. However, during the early stage of the infection (i.e., viremia) upon entering the nervous system, the SARS-CoV-2 binding to the ACE2 receptor is only limited to the gustatory and nasal epithelial cells. The activated CRS is still minimal at this stage, whereby patients may often recover after having only taste and smell impairments [104] (Fig. 2). However, past reports of SARS-CoV and MERS-CoV patients have demonstrated extensive CRS as the infection progresses, particularly in severely ill patients [105]. Moreover, pre-clinical animal study had shown that these viruses were able to aggravate the cerebral ischemic injury by triggering the cytokine cascade and potentiate the risk towards cerebral hemorrhage after administration of tissue plasminogen activator (tPA) [106]. Therefore, CRS seems to elevate the vascular permeability, edema, and widespread inflammation, and followed by MODS [107].

In COVID-19 patients, increased plasma pro-inflammatory cytokines such as IL-1, IL-6, IL-8, and TNF-α levels have been reported that may contribute to CRS onset in severe COVID-19 patients [102, 103]. IL-1 is produced by activated macrophages and dendritic cells in response to microbial stimuli that triggers fever, systemic inflammation, and tissues destruction. IL-1 is thought to exacerbate CRS, and phase 3 randomized control trial (RCT) of sepsis patients showed that IL-1 receptor antagonist anakinra (already approved for the treatment of rheumatoid arthritis) demonstrated survival benefits for patients with hyperinflammation [46, 108]. In severe COVID-19, the expression of IL-1α, IL-1β, and IL-1 receptor and their associated downstream signaling molecules were induced before respiratory function worsened. T cells activation was also observed, suggesting that IL-1 pathway exacerbated the disease through T cell-mediated cytotoxicity [109]. A retrospective study of severe COVID-19 patients with acute severe respiratory failure and systemic inflammation showed that those treated with anakinra demonstrated clinical improvements without deaths, decrease in oxygen requirements, and prolonged ventilation-free days [110]. Moreover, in severe COVID-19 patients, hyperinflammatory response has been reported in relation to ARDS and MODS [111]. In an independent retrospective cohort study of COVID-19 patients with moderate-to-severe ARDS and hyperinflammation, anakinra administration significantly improved the survival compared with patients receiving standard treatment [110]. Validation of anakinra as an anti-inflammatory treatment for COVID-19 is currently underway in RCTs (e.g., NCT04324021).

Numerous reports have also proposed IL-6 as an important mediator of CRS and severe respiratory failure [112, 113]. In acute inflammation, IL-6 is produced by activated macrophages and neutrophils [105, 114]. IL-6 promotes inflammatory cell infiltrate by rescuing T cells from apoptosis, promoting the maturation of T cells into effector T cells and inducing vessel permeability [114, 115], potentially contributing to organ damage by hyperinflammation and T cell-mediated cytotoxicity. Post mortem analyses of COVID-19 patients with ARDS complications showed hyperactivated cytotoxic T cells with concentrated cytotoxic granules [7]. A recent meta-analysis reported that mean IL-6 concentrations raised to nearly threefold higher in complicated versus uncomplicated COVID-19 patients and 9 out of 10 studies examined showed elevated IL-6 levels associated with worse prognosis [116].

Furthermore, observational and retrospective studies in COVID-19 patients have suggested the clinical efficacy of IL-6 blockade with therapeutic antibodies including tocilizumab [117], sarilumab, and siltuximab [118]. As such, RCTs are being conducted to examine the efficacy of IL-6 blockade in COVID-19 patients. In a press release on 29 July 2020, it was reported that the phase III COVACTA study (NCT04320615) involving 450 COVID-19 patients with severe pneumonia failed to meet its primary endpoint where tocilizumab administration did not confer improved clinical status compared with placebo. Nonetheless, tocilizumab-treated patients showed better trends in the duration of hospital stay and ventilator-free days. Moreover, COVACTA’s broad patient selection criteria and without apparently stratifying patients based on symptoms of hyperinflammation may have masked the potential benefits of tocilizumab [119], and the full trial data are eagerly awaited. The efficacy of tocilizumab is also being assessed in the RECOVERY late stage RCT of tocilizumab versus standard of care (NCT04381936) in over 850 COVID-19 patients.

It has been established that systemic inflammation confers debilitating effects on the brain, and the likely impact on the pathophysiology of CVD. Systemic inflammation induced by conserved pathogen-associated molecular patterns such as lipopolysaccharide and double stranded RNA in concert with pro-inflammatory cytokines could trigger inflammatory response from endothelium (i.e., endotheliitis) and cellular activation for a wider CNS inflammation [120]. Systemic TNF-α increase could induce the levels of IL-1β in the blood and brain, and that hypothermia and locomotor activity can be induced by IL-1β and IL-6 [121]. Recently, it has been shown that systemic inflammation is associated with CSVD. The severity and progression of CSVD are strongly associated with systemic inflammation characterized by increased circulating IL-6 and its production by monocytes [122]. Furthermore, higher serum levels of IL-1α and IL-6 were significantly associated with the primary outcomes of CSVD in both univariable and multivariable analysis adjusted for age, sex, and CSVD radiological markers, and both ILs levels had the strongest association with recurrent stroke in the disease [123].

In COVID-19 patients, the prolonged exposure to physiological stress and hyperinflammation during CRS may contribute to various neurological symptoms (i.e., neurocognitive, and neuropsychiatric) [124]. Hence, alongside the systemic inflammation is heightened oxidative stress, and both responses have been associated with the pathogenesis of CSVD as in arteriosclerosis [125]. Oxidative stress-related species such as reactive oxygen species (ROS) and reactive nitrogen species (RNS) contributed to cerebral vascular oxidative stress by elevating the inflammatory response that influence the progression of clots or thrombus, increase pro-inflammatory cytokines (i.e., IL-6, IL-8, TNF-α, monocytes chemoattractant proteins-1 [MCP-1]), endothelial function, and increased expression of vascular endothelial adhesion molecules (VCAM-1) and intracellular adhesion molecules (ICAM-1) [126]. Subsequently, elevated levels of RNS and ROS have been associated with oxidative stress-mediated cell migration and proliferation, DNA damage, necrosis and apoptosis, cellular autophagy, endothelial dysfunction, and endoplasmic reticulum stress [127]. Furthermore, following overproduction of pro-inflammatory cytokines is the activation of transcription factors (i.e., nuclear factor kappa B [NF-κβ] and/or nuclear factor (erythroid-derived 2)-like 2 [Nrf2]) and signal transduction cascades [128] that elevate the release of cytokines and chemokines that further enhance inflammation [129]. However, nitric oxide (NO) release by ECs inhibits the expression of NF-κβ and adhesion molecules; hence, NO serves as a crucial anti-inflammatory factor and important for vascular vasodilation. However, this ability is diminished following ECs damage with the systemic inflammation [130].

Additionally, ROS may act on the ECs-induced inflammation through the disruption of inter-endothelial junction, gap formation, actomyosin contraction, and altered phosphorylation or expression of junctional adhesion molecules [131, 132], leading to endotheliitis. Therefore, COVID-19 endotheliitis has been proposed to cause compromised microvascular structure and function in various vascular beds, resulting in the clinical sequelae in COVID-19 patients [43]. Moreover, the released cytokines from the induced inflammation of ECs through extracellular matrix (ECM) degradation is followed by BBB breakdown [133]. Besides endothelium, there exists crosstalk among cellular components of the BBB such as pericytes, astrocytes, and oligodendrocyte precursor cells (OPCs) that are likely to be involved in the microvascular damage as precursors for the onset and progression of CSVD [134, 135]. In fact, mice infected with coronavirus had developed acute demyelination with the involvement of microglia, ECs, and astrocytes [136]. In relation to this, reduced white matter integrity due to changes in oligodendrocytes has been shown in CSVD, whereby the ECs-OPC signaling was compromised that altered the ECs’ ability to secrete the releasing factor crucial for the growth and survival of OPCs, which in turn caused oligodendrocytes damage [137]. An increased BBB damage and permeability further induced the degradation of basement membrane of ECs and accumulation of ECM components leading to stiffening of vessel walls [138]. Furthermore, the BBB damage will further intensify as the deposition of blood components such as platelets, microparticles, and fibrin increased after BBB breakdown. Several studies supported that changes in walls of small vessels in the brain due to BBB breakdown would lead to ischemic events classified as WMHs, lacunar infarcts, and CMBs, with and without COVID-19 infections [87, 139–141].

In addition, obesity as part of the metabolic syndrome is thought to elicit low-grade inflammation in relation to ARDS [142] and is associated with an elevated cytokine IL-33 level that mediated the stimulation of pro-coagulant tissue factor (TF) release by ECs [143]. Hence, in relation to COVID-19, these factors increased the likelihood of obese patients to develop a more severe COVID-19 while at risk of stroke [144]. Collectively, these data indicate that pro-inflammatory cytokines and oxidative stress are involved in the pathogenesis and severity of CSVD. As CRS is one of the hallmarks of critically ill COVID-19 patients, it is plausible that administration of immune-suppressive medications such as IL-1 or IL-6 blockade with therapeutic antibodies and antioxidative agents may also mitigate the risk of CSVD in COVID-19 patients.

### Hypercoagulation and Cerebral Microthrombosis

In the later stage of COVID-19 infection (i.e., pneumonic and/or hyperinflammatory stage), SARS-CoV-2 is reported to further heighten the activation of uncontrolled cytokines release leading to hyperinflammation, elevation of CRP, ferritin, and D-dimer levels [104] (Fig. 2). Systemic pro-inflammatory factors such as ILs, TNF-α, and CRP are responsible for the primary molecular events elicited by abnormal coagulation or hypercoagulable state [104, 145, 146], and SARS-CoV-2 is thought to foster a pro-inflammatory microenvironment and induce prothrombotic state leading to thrombogenesis, formation of blood clots, and small or large vessel occlusion [76, 147]. Besides, the elevated immune response may also lead to vasculitis in nerves and muscles, alongside with immune-mediated peripheral, cranial nerves and/or muscle injury [104].

In general, the coagulation process or pathway serves to maintain hemostasis or to control bleeding, promote healing, and prevent spontaneous bleed [148]. The coagulation pathway is controlled by certain naturally occurring inhibitory elements or anticoagulants such as protein S, protein C, antithrombin, and tissue factor pathway inhibitor (TFPI) that control and limit the formation of clot to prevent propagation of thrombus/microthrombus or further thrombosis/microthrombosis [148]. Altered pro-coagulant properties of such coagulation factors would stir imbalance in the pathway, either with increased or decreased activities of a given factor [149]. Generally, the thrombogenic elements of coagulation factors are produced from two sites: the vessel wall (i.e., TF, exposed endothelium, and collagen) and the circulating elements (i.e., platelets, platelet activating factor, prothrombin [factor II], fibrinogen [factor I], von Willebrand factor [vWF], and numerous clotting factors). Certain events such as physiological disturbance, blood abnormalities, infection, elevated pro-inflammatory cytokines activities, and disturbance in the primary hemostasis (i.e., platelet plug formation at the insulted site of exposed ECs of the vessel wall) would result in the imbalance of the coagulation system, hence termed as coagulopathy [150, 151]. Thus, in relation to COVID-19 infections, an altered systemic coagulation cascade in microcirculation can be activated at early disease process, and platelet activations are the main player in microthrombi/clots formation and its plausible impact on the pathomechanism of CSVD (Fig. 1).

COVID-19-associated coagulopathy reported among COVID-19 patients warrant further investigation as SARS-CoV-2 has no known direct intrinsic pro-coagulant effect [152]. Moreover, emerging evidence has shown that SARS-CoV-2 can cause microvascular, arterial, and venous thrombosis through ACE2 receptor on the ECs and smooth muscle cells (SMCs) hence potentiate organ injury [153]. In addition, SARS-CoV-2 may also invade ECs of the cerebral arterioles eliciting direct and/or immune-mediated injury without compromising systemic response and hence partly explains why COVID-19 patients with no systemic symptoms are at risk of cerebral vascular injury and cryptogenic stroke [85]. In addition, hypercoagulability in COVID-19 patients has been reported to be associated with elevated acute-phase reactant levels including CRP and fibrinogen, thus being used as biomarkers with prognostic values [154, 155]. Abnormalities in coagulation system are frequent among fatal cases of COVID-19 including shortened aPTT and prolonged PT in coagulation cascade [64, 156]. Moreover, FDPs such as D-dimer and other FDPs are the most widely used and direct prognostic biomarkers for COVID-19 severity and often in fatal cases compared to non-severe patients with a higher plasma D-dimer [45, 155, 157–159] and, thus, posed an increased risk towards CSVD-associated microthrombosis [93].

As elaborated previously, the viral infection may potentiate the innate immune response such as systemic inflammatory activation. The response can activate the coagulation cascade followed by the generation of thrombin or generally referred as thrombo-inflammation or immuno-thrombosis as part of crucial communication components among cellular and humoral amplification pathways [160, 161]. Furthermore, infection-based inflammatory response mediates coagulation cascade through multiple pro-coagulant pathways. In this case, virus-derived polyphosphates may induce the activation of platelets and factor XII in coagulation pathway, thus amplifying the downstream pro-coagulant response especially the intrinsic pathway of coagulation cascade [162]. Additionally, elevated levels of viral infection-based inflammatory biomarkers such as pro-inflammatory cytokines instigate the activation of vascular ECs and endothelial injury/dysfunction that further promote the thrombo-inflammation [163]. Another important component that activates and enhances the contact and prothrombotic pathway respectively is the cell-free DNA and histones neutrophil extracellular traps (NETs) that present and propagate as part of the intravascular thrombi, hence triggering the generation of thrombin [163, 164].

SARS-CoV-2 infection also can directly cause cellular activation (e.g., ECs, neutrophils, monocytes, macrophages, T cells) that provoke further pro-inflammatory cytokines release and hence increase the disruption of endothelial function and integrity, followed by the release of vWF, DIC, and upregulation of extracellular particles formation (i.e., P-selectin, and intercellular adhesion molecules, ICAM-1) [155, 165, 166]. In addition, NETs have also been identified in early phase of COVID-19, suggesting that activated neutrophils may also play a significant role in the formation of microthrombi [167, 168]. NETs contribute to initiating the extrinsic and common pathway through elevating the activation of TF, factor XII, and platelets [169]. Therefore, CRS, elevated level of activated neutrophil, formation of NETs, and cellular activation products collectively serve as potential microthrombogenic markers of SARS-CoV-2 infection and circulating cells aggregation, resulting in generation of intra-arterial thrombus or microthrombus which is likely to contribute to the pathogenesis of arterio-micro-thrombotic diseases such as CSVD.

Of note, severe complications of COVID-19 are characterized by ARDS and pneumonia that rapidly progress to MODS. COVID-19-associated ARDS is related to endothelial dysfunction-associated vascular micro-thrombotic disease, which also involves MODS that hastens mortality in COVID-19 patients [170]. It has been proposed that these complications are secondary to COVID-19-induced endothelial dysfunction that cause the imbalance between limited vWF-cleaving protease and elevated exocytosis of vWF from ECs [171, 172]. Moreover, ECs-derived ultra large vWF (ULVWF) multimers enable the recruitment of platelets and mediate microthrombogenesis within microvasculature and eventually initiate the production of large microthrombi [172, 173]. Furthermore, the microthrombi formed can be rapidly activated and further elevates the aggregation of platelets and platelets-derived microparticles (i.e., P-selectin) inducing leukocytes aggregations. These aggregates or microclots will then dislodge from ECs luminal surface into the circulation and may occlude smaller vessel distally [172].

In the case of COVID-19, several studies had reported the presence of blood clots in COVID-19 patients with cerebral ischemia in both cerebral arteries and veins [57, 62]. Xiong and colleague supported that CRS may trigger hypercoagulation cascades that lead to formation of large and small blood clots [174]. In SVD, the activated platelets and microthrombi formation would in general initiate the narrowing of the arterial wall and upregulate the proliferative arterial wall changes [175]. Platelet aggregation is also known to result in the release of vasoactive substance resulting in SMCs constrictions, hence narrowing the arterial wall [176, 177]. Moreover, microthrombi consist of white thrombi of aggregated fibrin and platelets that narrowed the arterial lumen as evident by the intraparenchymal small vessel microclot/microthrombosis found in cerebral ischemia or infarcts [178, 179]. Microthrombosis-mediated cerebral microcirculatory dysfunction has been suggested as an outcome of intraparenchymal small vessel dilation that compensated the reduction in perfusion from peripheral pressure of larger arteries. This occurred as a small vessel trying to optimize the dilation process to maintain the CBF following the arterial lumen narrowing [180].

Therefore, it is plausible to deduce that COVID-19 elicits a micro-thrombotic disease manifestation, consisting of large amounts of circulating complexes of ECs derived microthrombi, filtered or embolized in microvascular bed with a potential micro-thrombotic occlusive impact, serving plausible roles in the onset and progression of CSVD if left untreated.

### SARS-CoV-2 Proliferation Mediated Hypoxia Injury

It is well known that initial SARS-CoV-2 infection infiltrates the pneumocytes resulting in hypoxia that in turn increases the risk of CVD among COVID-19 patients [52]. This is because once the virus proliferates in the lung tissues, subsequent insults are worsened by acidic interstitial inflammatory exudation and diffuse alveolar damage [52, 181]. The latter is characterized by accumulations of hyaline along the wall of alveoli (membrane made from dead cells, proteins, and surfactant) which disrupts the alveolar gaseous exchange [181]. Consequently, these trigger the elevation of mitochondrial anaerobic metabolisms in the brain cells causing CNS hypoxia [181]. Besides, the accumulation of acids also eventually leads to altered CBF, cerebral vasodilation, and ischemia, whereby further unabated hypoxia will cause cerebral microcirculation disturbance in the brain parenchyma structure and function [181]. Inevitably, this hypoxia potentially triggers the onset of cerebral ischemia (i.e., ischemic stroke) especially in individuals with concomitant CVD risk factors. In COVID-19 patients, this hypoxic drive is likely to inflict further brain damage with an increased risk of fatality [182].

### Others: Myocardial Injury and Cerebral Hypoperfusion

Another potential indirect infection is through cardio-embolism from SARS-CoV-2 associated with a myocardial injury. SARS-CoV-2 proliferation-associated CRS and subsequent immune responses may lead to myocardial injury by reducing the coronary blood flow, oxygen supply, and destabilized coronary plaques and elevating microthrombogenesis [182]. Furthermore, single or combination of systemic factors such as pro-inflammatory cytokines release, hypercoagulability, and complement-mediated microvascular thrombosis may also lead to endothelial dysfunction as seen with marked increases in the levels of pro-inflammatory markers and D-dimer [49, 76, 183]. The endothelial dysfunction can be further aggravated by COVID-19-mediated RAS disruption that may result in a secondary CBF dys-autoregulation [75], leading to cerebral hypoperfusion. This has been shown in CADASIL patients with COVID-19 where CSVD lesion manifested at the cerebral internal border zone, a region that is prone to hypoperfusion [184]. Locatelli and colleagues posited that CADASIL patients may suffer from a chronic cerebral hypoperfusion mainly from the disruption in the myogenic component of CBF autoregulation where SMCs become constricted or dilated in response to changes of transmural pressure [184].

## SARS-CoV-2 as Potential Risk for Circulating Microparticles Release

The mechanism that causes the cerebral ischemia or the onset and progression of CSVD in patients with COVID-19 remains elusive at present, although clues are linked to hyperinflammation and hypercoagulability [50]. A recent review on COVID-19 and neurovascular complication described that SARS-CoV-2 can remain in the human systemic circulation or in neurons without toxicities [59]. The abnormal proteins misfolding and aggregations caused by SARS-CoV-2 may trigger future NDD among COVID-19 patients who have recovered and discharged from intensive care [185]. The initial trigger is associated with cytokine storm, cellular activation, and hypercoagulability that inflict damages to cerebral vascular (small/large) and BBB, which in turn may result in long-term neurological sequelae. Therefore, in this section, we put forward the proposition that COVID-19 infection may also potentially mediate another detrimental pathomechanism for COVID-19-related CSVD, i.e., the extracellular circulating microparticles (MPs).

### Overview of Extracellular Circulating MPs

Recently, there is an increasing interest in the identification and quantification of cellular debris such as extracellular vesicles (EVs) as biomarkers to study the natural history of development and progression of several diseases including cardio-cerebrovascular disease, cancer, metabolic disease, and blood disease (i.e., sepsis). EVs, also known as cell-derived particles or extracellular particles (EPs), are anucleate phospholipid bilayer membrane released from cells with encapsulated particles such as proteins, lipids, nucleic acids, and metabolites. Flow cytometry is the most widely used method and has major advantages over the other techniques in that each EVs (and its subtypes) is interrogated individually for the identifications and quantification based on antigen expression [186]. To date, there is no consensus in the nomenclature of EVs of different sizes, composition, and origin [187]. In general, EVs can be classified into three standard categories that include exosome (the smallest EVs: 30–100 nm in diameter), microparticles (MPs) or ectosome (100 nm–1 µm in diameter), and apoptotic bodies (large membrane blebs: ≤ 5 µm in diameter) [188, 189].

**Fig. 4:**
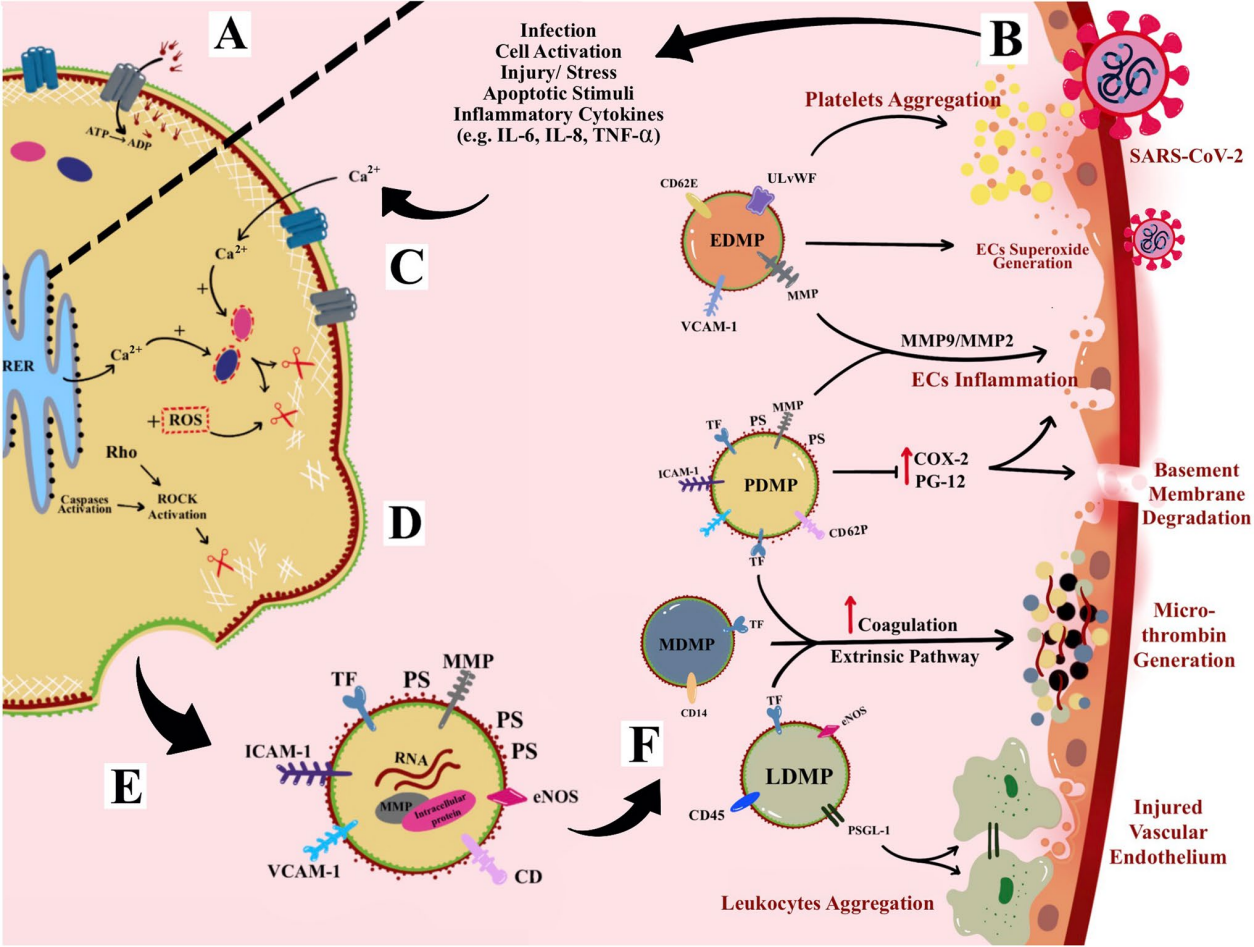
Microparticles (MPs) formation and mechanism of action. **A** Active translocase transporting phosphatidylserine (PS) from outside to inside layer through adenosine triphosphate (ATP)-dependent manner. **B** Cellular activation due to infection or other cellular stressor such as increase cytokines and apoptotic stimuli. **C** The activation causes an increase in intracellular cytosolic calcium release by stress endoplasmic reticulum (ER) and acquired from extracellular space and hence activates enzymes calpain and gelsolin that cleave cell membrane cytoskeleton. **D** The cleaved cytoskeleton causes inactivation of translocase and, hence, induces phospholipid “flip-flopping.” **E** Externalization of PS produces MPs that bring their parent surface molecules and protein antigens. **F** MPs production can trigger series of micro-thrombotic cascades. For example, leukocytes-derived MPs (PDMPs) contain P-selectin glycoprotein ligand-1 (PSGL-1) on its surface that enables leukocytes-endothelial cell (ECs) adhesion. Most MPs contain tissue factor (TF) associated with an increase in the extrinsic coagulation cascade and production of microthrombus. In fact, PDMPs and endothelial cell-derived MPs (EDMPs) may bring pro-inflammatory antigens such as matrix metalloproteinase (MMP) that can cause endotheliitis. EDMPs also possess ultra-large von Willebrand factor (ULVWF) that further assists in the recruitment and aggregation of platelets on endothelium

MPs are anucleate, small, and membrane-enclosed EPs [190–192]. Ranging from 0.1 to 1 μm in diameter, MPs are derived from direct deformation of cell plasma membrane and cell membrane phospholipids exocytic blebs that are released from the cell surface by proteolytic breakdown of the cytoskeleton due to various triggered mechanisms such as virus infection, cellular activation, oxidative stress, inflammation, injury, or apoptosis. In this context, factors such as different agonists, thrombin, serine proteases, collagen, pro-inflammatory cytokines, and physiological shear stress contribute to cellular activation and further promote the secretion and aggregation of MPs [191, 193–195]. In addition, during apoptosis, the apoptosis-induced MPs release is stimulated by the caspase-mediated Rho effector protein, the “Rho-associated” coiled-coil-containing protein kinase 1 (ROCK-1), as well as by thrombin and TNF-α [196]. General mechanism of MPs formation and its mode of action is described in Fig. 4.

Moreover, MPs are heterogeneous and can be produced from multiple sources (or parental cells) within the blood circulation such as platelets, erythrocytes (red blood cells, RBCs), leukocytes (white blood cells, WBCs), monocytes, ECs, and SMCs [197]. Also, MPs can be found in various body fluids such as saliva, urine, bile, CSF, and synovial fluid [198]. MPs are identified by the presence of pro-coagulant cell surface marker phosphatidylserine positive (PS^+^), although recent evidence had suggested instances of PS negative (PS^−^) [199]. Moreover, in the blood circulation of healthy individuals, MPs are present in low levels, while 70–90% of MPs are represented by platelets-derived MPs (PDMPs) that could play a role in various disease pathologies [200]. MPs composed mainly of cytosol, enclosed by globose phospholipids bilayer, whereby their cytosol may include RNAs (i.e., non-coding small interfering ribonucleic acid [siRNAs], messenger RNA [mRNA], and micro-RNA [miRNAs]) [201, 202], enzymes, and cytoskeletal proteins of their parental cells, but are anucleate and lack synthetic capacity. However, to date, there is no evidence of deoxy-ribonucleic acid (DNA) presence in MPs luminal space, although a trace of DNA has been found in exosomes and apoptotic bodies [203].

MPs carry their own parental membrane proteins or markers which are used to identify their cell of origin or subpopulations. For examples, cluster differentiation 41 (CD41) is used to identify PDMPs, CD235/ CD235a for RBCs-derived MPs (RDMPs), CD31/CD146 for ECs-derived MPs (EDMPs), and CD45 for leukocytes-derived MPs (LDMPs) [204]. Interestingly, PDMPs transport over 40 membrane integral protein or glycoprotein characteristic of platelets, such as integrin β1 (CD29), αIIbβ3 (CD41), and P-selectin (CD62P). PDMPs and EDMPs also contain pro-invasive/pro-inflammatory matrix metalloproteinase proteins (MMPs-2/9). Most of these proteins serve as adhesion molecules that stimulate the EVs internalization by these cells [200], Meanwhile, RDMPs are the smallest (~ 0.15 μm) compared to other cell-derived MPs whereby their surface consists of residual hemoglobin (20% from parent RBC) [205, 206].

#### MPs Roles in Coagulation and Microthrombosis

MPs’ pro-coagulant and prothrombotic properties are partly due to their abilities to bind to sub-endothelial matrix (and its components), adhesion with soluble/non-mobile fibrinogen, as well as co-aggregation with platelet aided by a complex process involving glycoprotein (GP-IIb/ GP-IIIa) [207]. As mentioned, PS presence on MPs surface acts as pro-coagulation factors for assembly and binding agent or proteins in coagulation cascade that may lead to a prothrombotic state [193]. PS binds to hematopoietic-derived clotting factors through electrostatic interactions between phosphate groups in phospholipids and Ca^2+^ in gamma-carboxyglutamic (GLA) domain of clotting factors [208]. Factors VII, IX, and X and prothrombin are the clotting factors that contain GLA domain. Therefore, the recruitment of PS-bearing MPs and clotting factors promotes the aggregation of platelet and synthesis of fibrin which confer the propensity for the formation of microthrombus [209] that could play a plausible role in CSVD pathogenesis.

In fact, in vitro study had shown that even low levels of MPs (i.e., PDMPs and EDMPs) could induce the generation of microthrombus [210]. If compared to activated platelets (i.e., parental cells), PDMPs surfaces possess up to 100 times higher pro-coagulant properties and higher affinity binding sites for activated coagulation cascade [211]. It appears that PDMPs serve as a precursor for microthrombus formation by providing catalytic surfaces for the prothrombinase enzyme complex (i.e., involving factors IXa, Va, VIII, and Xa) [209].

Moreover, MPs also transport pro-coagulant surface TF, where MDMPs had been shown to bring an active TF that potentially elevated the extrinsic pathway involving factors VII, VIIa, IX, and X in coagulation cascade [212, 213]. As for LDMPs that express P-selectin glycoprotein ligand-1 (PSGL-1) and platelet P-selectin on their surfaces, these elements further aid the aggregation of TF bearing leukocytes at the site of vascular or microvascular injury [214]. In fact, the formation EDMPs had also been associated with elevated levels of endothelial dysfunction markers such as plasminogen activator inhibitor-1 (PAI-1) and elevated the pro-coagulant activity and prothrombotic state. This was due to EDMPs that contained the expression of ULVWF multimer that enabled EDMPs to induce strong platelet aggregations [215]. Thus, TF-bearing MPs may play an important role in micro- and macro-thrombus formation. In a different but related context, a study had shown that tumor cells-derived MPs bearing both PS^+^ and TF can be utilized as reliable biomarkers to determine the risk of venous thrombosis in cancer patients [194] (Fig. 4).

#### MPs and Inflammation

The release of MPs into the circulation that ensued cell/tissue inflammation can further aggravate the inflammatory activity [216]. MPs can affect microcirculation by potentiating the production and expression of pro-inflammatory cytokines, chemokines, and ICAM-1 [217] (Fig. 4). In vitro study had shown that ECs and monocytes interaction with PDMPs able to elicit the de novo expression and production of inflammatory molecule/agent such as cyclooxygenase (COX-2) and prostacyclin (PG12), respectively [218]. Another in vitro study had also shown that EDMPs are involved in the up-regulation of E-selectin, ICAM-1 and VCAM-1, and induction and release of pro-inflammatory cytokines (i.e., IL-6 and IL-8) [219].

#### MPs and Cell Signaling

Alongside with MPs pro-coagulant and pro-inflammatory abilities, they also serve as mediators for cell–cell interactions and signal delivery between cells. Since MPs bring along specific parental membrane receptors, cytosolic proteins, and RNAs, they can stimulate certain target cells to transform and communicate with microcirculation, in ways programmed by these surface markers [220]. For example, PDMPs can stimulate B cells to synthesize specific antibodies such as IgG by delivering CD154 IgG [221]. In addition, PDMPs assisted in monocytes to ECs interaction through ICAM-1 and hence elevated chemotaxis of monocytoid cells [218]. Furthermore, it has been shown that once PDMPs form a close contact with neutrophil, it can bind and increase neutrophil aggregations and elevate neutrophil phagocytic activity [222]. Moreover, MPs can be phagocytosed by certain cancer cells and hence stimulate the cell to induce the expression of mRNA for the pro-invasive MMP-9 and upregulate the adhesion to ECs in order to activate the ECs leading to the endothelium dysfunction. The EDMPs that expressed proteases proteins such as MMP-9 and MMP-2 enabled the invasion towards vasculature through disruption of basement membrane [223, 224] (Fig. 4).

#### MPs and Related Clinical Syndrome

It is well accepted that the elevated levels of MPs in blood circulation are reflective of their multi-faceted roles; for example, higher level of MPs was found in hypertensive patients [225], abdominal obesity [226], myocardial infarction [227], tumor progression and metastasis [228], atherosclerosis [229], and cardiopulmonary bypass patients [211]. Previous in vitro studies also showed that elevated T lymphocytes-derived MPs induced arterial endothelial dysfunction (i.e., reduce expression of NOS) in immune-compromised states [230, 231]. Moreover, MPs can also contribute to acute lung injury and inflammatory airway disease [232], with an elevated level of MDMPs being associated with upregulated pro-inflammatory IL-8, ICAM-1, MCP-1, and superoxide anion production and activation of NF-κβ in monocytes [232, 233]. Besides, elevated EDMPs level has been reported to correlate with severity of endothelial dysfunction in heart diseases, i.e., coronary artery disease and acute coronary syndromes with worst clinical outcomes [192, 234, 235].

In the case of nervous system disorders, MPs had been shown to contribute to both pro- and anti- inflammatory responses in inflammation-mediated NDD including Parkinson’s disease (PD), AD, amyotrophic lateral sclerosis (ALS), and dementia [236]. CNS-derived MPs had been shown to circulate in peripheral circulation and proposed to influence the cerebral immune status by transferring peripheral pro-inflammatory molecules to CNS [92, 237, 238]. Recent evidence also suggested that MPs-mediated release of pro-inflammatory cytokines, miRNAs, and microbial by-products are associated with the onset, progression, and resolution of inflammation-based cerebral injury and NDD [239, 240]. Therefore, these associations confer circulating MPs as pertinent clinical targets and potential biomarkers of disease onset and/or progression, including that for CSVD.

### COVID-19 and MPs

Viral infections are known to give rise to pathologic consequences such as thrombotic and hemorrhagic complications as seen with CVD [241, 242]. However, reports on the involvement of MPs related to COVID-19 remain scarce. Thus, bodies of evidence that may implicate MPs in the setting of COVID-19 infection are highlighted in this section.

As one of the main activators for coagulation, TF is present on the surface of certain circulating cells such as monocytes and ECs. It can also be expressed by the pathogens and inflammatory cells [243]. Moreover, the activation of ECs in viral infections may interfere in normal coagulation and fibrinolytic system, both directly and/or indirectly [244]. Viral infection initiates pro-inflammatory CRS and hence inflammation that causes imbalance in coagulation systems, resulting in coagulopathy such as thrombosis and/or hemorrhage [245]. Furthermore, with this imbalance, further microvascular thrombosis may occur that could lead to MODS and DIC [246] as seen in an infectious disease such as malaria previously reported with a higher level of MPs [247].

A higher risk of arterial thrombosis has been documented in critically ill patients with COVID-19-based hypercoagulation, where patients developed thrombi in the lungs [50, 248]. However, it is suggested that thrombosis may well occur from the early phase of COVID-19 infection and worsened as the disease progressed [50]. Moreover, the formation and stimulation of pulmonary clots and NETs respectively halt the viral infection and further inflammation at vascular endothelium of the lungs alveolus even at an early stage [249]. These microthrombi may disseminate into the peripheral circulation and eventually aggregate to become larger thrombi within the background of untreated inflammation or CRS. Furthermore, it is known that COVID-19-based hypercoagulability are not limited to the lungs only but has been observed in the gastrointestinal tract (GIT), cerebrovascular and coronary ischemia, and even in lower limb [43, 248, 250], hence suggesting that the initial microthrombi produced in the lung can potentially embolize in microcirculation to settle and accumulate in distant organs.

Following viral invasion, the vascular endothelium served as the main trigger site as the general interface between immune and hemostatic systems [43]. ECs damage or activation is initiated when viruses bind to ACE2 receptor in type II pneumocyte of human lung epithelium (i.e., AT2) and myocardium, where these receptors are also highly expressed in arterial ECs [21] (Fig. 1). Once ECs are activated, they can promote an acute inflammation followed by hypercoagulation, and hence thrombosis. However, under a pulsatile shear stress condition, the increment in the ACE2 expression could promote the production of NO and, thus, reduce the inflammation and proliferation in vascular ECs [251]. Consequently, COVID-19 patients with heart failure or myocardial disease become more vulnerable to further infections [102]. One of the main targets of SARS-CoV-2 infection is the pericytes that surround the outer layer of ECs of capillaries and venules, reflecting the likelihood of capillary ECs dysfunction and microcirculation disturbance [102]. It has been proposed that during CRS, plasma membrane remodeling resulted in the exposure of the pro-coagulant PS, hence implicating MPs shedding in the general pathomechanism of ECs dysfunction. Moreover, pro-inflammatory factor such as TNF-α may also induce the production of ACE2-harbored EDMP in microvascular ECs [252]. Hence, it is plausible to posit that EDMPs-bearing ACE2 may systemically embolize from the site of formation (i.e., lung) to the distant target (i.e., brain), depositing the virus and further aggravating disease complications.

SARS-CoV-2 infection involving ECs could lead to endothelial dysfunction or endotheliitis. Therefore, measuring and enumeration of specific markers such as the selectins and MPs could prove to be beneficial to study the disease onset and progression for future prevention and therapeutic measures [50, 253]. Furthermore, MPs produced by ECs damages as a result of viral infection can further stimulate the elevation of pro-inflammatory cytokines (i.e., IL-1, IL-6, IL-8, and TNF-α) [254]. Consequently, elevated levels of MPs can serve as a positive feedback to disease manifestation. Recently, it had also been shown that the expression of pro-inflammatory cytokines such as IL-6 and IL-8 following MPs formation can exasperate COVID-19 and been proposed to MPs (and its subpopulation) to serve as panel of markers for COVID-19 onset, progression, and severity [250].

## Proposition and Potential Implications for COVID-19 related MPs and Risk for CSVD

To date, limited studies are available to implicate the role of MPs in micro-thrombosis [194] and CSVD. However, there are evidence that MPs levels are increased in patients with cardiovascular diseases and related risk factors, including acute coronary syndromes, diabetes, hypertension, and hypertriglyceridemia and the spectrum of CSVD [193, 255–257]. Table 1 summarizes the potential associations of CSVD with MPs subpopulation from published literature to date.

**Table 1:** Association of CSVD with MPs subpopulations

CSVD correlates/findings	Changes in MPs level based on their surface markers/cluster differentiation (CD)	MPs—parent cells
• One hundred ten patients (mean age, 71.1 ± 7.9 years) with acute-phase cerebral infarction, 34 with small vessel occlusion [258]	• Higher level of CD61^+^, CD62P^+^ (P-selectins), and CD42^+^/CD42a (glycoprotein IX)	Platelet-derived microparticles (PDMPs)
• Cerebral infarction attributable to vasospasm in 20 elderly subjects (mean age: 52.2 years old), suggesting the consequences of microthrombosis [259]	• Higher level of CD41^+^ and CD41^+^/annexin^+^	
• Forty middle-age subjects (mean age: 44.4 ± 12.2 years) with metabolic syndromes [260]		
• Silent brain infarct in subcortical white matter in 15 male healthy obese subjects and 50 male obstructive sleep apnea subjects (more prevalent) [261]	• Higher level of CD40L and soluble P-selectin	
• Middle cerebral artery occlusion in a rat model with cerebral infarction [262]	• Higher level of CD41^+^	
• In individuals with micro-embolic cerebral ischemia and associated with recent cerebrovascular events as seen in DWI [148]	• Increase total PDMPs	
• Forty-one elderly individuals with mild, moderate to severe ischemic stroke [263]	• Higher level of CD105^+^/PS^+^ and CD144^+^ (marker for apoptotic-derived MPs)	Endothelial-derived microparticles (EDMPs)
	• Higher level of CD54^+^ (marker for ECs activation)	
• One hundred twenty-nine elderly individuals (68 with acute ischemic stroke [mean age: 63.59 ± 13.33]) [264]	• Higher level of CD144^+^ and CD31^+^ (marker for apoptotic-derived MPs)	
	• Higher level of CD62E (marker for cellular inflammation)	
• One hundred one middle-age individuals with metabolic syndrome (with and without chronic heart failure), suggesting the relevance to neurohumoral and inflammatory activation [192]	• Higher level CD31^+^/annexin^+^ and lower CD62E^+^	
• Eighteen individuals with subcortical and periventricular subcortical lesion [265]	• Higher level EDMP bearing VCAM-1 and soluble P-selectin	
• Related to higher WMHs and the progression of brain atrophy in individuals (n = 534, 4 years follow-up) with vascular disease manifestation [256]	• Higher level CD14 (monocytes-derived MPs)	Leukocytes-derived microparticles (LDMPs)
• An increased risk of arteriothrombotic stroke with individuals with obstructive sleep apnea [266, 267]	• Higher level CD45^+^ and CD45^+^/annexin^+^ (mostly leukocytes-derived MPs)	
• Cerebral infarction attributable to vasospasm in 20 elderly subjects (mean age: 52.2 years), suggesting the consequences of microthrombosis [259]		
• In individuals with cardiometabolic risk factors such as T2DM and dyslipidemia [268]	• Higher level of CD4^+^/TF^+^ (lymphocytes-derived MPs)	
• Seventy-six elderly individuals with ischemic cerebrovascular diseases [269]	• Higher level of CD45^+^, CD14^+^, CD4^+^, and CD15^+^ (granulocytes-derived MPs)	
• Cerebral infarction attributable to vasospasm in 20 elderly subjects (mean age: 52.2 years), suggesting the consequences of microthrombosis [259]	• Higher level of CD235^+^ and CD235^+^/annexin^+^	Red blood cells-derived microparticles (RDMPs)
• Induced cerebral neuronal cell death in vitro [270]	• Higher level of CD47*	

*CD* cluster differentiation, *CSVD* cerebral small vessel disease, *DWI* diffusion weighted imaging, *ECs* endothelial cells, *MPs* microparticles, *T2DM* type 2 diabetes mellitus, *WMHs* white matter hyperintensities

^*^Least data on its association with CSVD, compared to other MPs subpopulation described above

MPs can be formed locally or distally, and then aggregated to initiate microthrombi cascade in cerebral microvasculature (i.e., end arteries). As the microthrombogenic MPs embolized and settled at the lumen of cerebral microvasculature, they may increase the vascular tone, impair vascular relaxation, stimulate angiogenesis, and stimulate cells to produce cytokines and other inflammatory mediators as well as mediate intercellular interactions. They may also activate the formation of free radicals [261, 271]. MPs are capable of these pro-coagulant effects due to the fact that they bear functionally bioactive phospholipids and cyto-adhesion molecules, such as PS and pro-coagulant TF known to play major roles as cellular activators of the coagulation cascade [194]. Moreover, the formation of MPs might contribute to the disorganization of the proper function of endothelial structural layers. For example, Martinez et al. (2011) [272] showed that endothelial dysfunction caused by MPs lowered the production of NO and thus induced vascular inflammation that potentially contributed to the prothrombotic state within the arterial wall and propagated atherosclerosis a hallmark of endothelial dysfunction. Besides, this dysfunction had been demonstrated to involve shedding of EDMPs that expressed platelet-ECs adhesion molecule-1 (i.e., CD31) and implicated in ischemic stroke subtypes [273]. Thus, we proposed that a targeted enumeration of MPs through peripheral venous blood in clinical setting may serve as supportive biomarker for early detection and/or prevention of CSVD, particularly among at-risk, asymptomatic individuals.

Hypercoagulability, hyperinflammation, and endotheliitis are the three prominent features of COVID-19 infection, markedly so in severe cases which affect prognosis by prothrombotic events including CSVD. Schreiber et al. (2013) [274] also proposed another common pathomechanism of CSVD related to the disorganization of arterial segmental walls and luminal narrowing. These arose due to accumulations of MPs alongside cholesterol crystals that caused arteriolosclerosis, which may result in hypoperfusion that accompanied infarcts and WMHs [274, 275]. Besides, these features also pose added risks to the well-known features for critically ill patients (as in severe COVID-19 cases) with respiratory failure, mechanical ventilation, central venous catheter, and prolonged immobilization. Notwithstanding, the precise underlying pathomechanisms for significant cases with thrombosis associated with COVID-19, despite prophylactic and therapeutic measures, remain elusive. Hence, evidences presented here on circulating MPs offer potential implications as prognostic markers particularly in severe COVID-19 cases who are likely to be more vulnerable to MPs-mediated hypercoagulation. Moreover, in less severe or early stages of COVID-19 cases, MPs could serve as monitoring biomarkers in setting of low-grade inflammation especially those related to asymptomatic CSVD and cryptogenic stroke subtype. Essentially, such a monitoring could help mitigating the risk of such patients to develop or progress to a more severe condition.

Furthermore, there is growing body of evidence indicating the higher prevalence of COVID-19-associated small vessel stroke (i.e., cryptogenic stoke) to implicate COVID-19 infection in novel small vessel stroke mechanism. For example, a recent meta-analysis study reported a fivefold increment in in-hospital mortality among a patient with COVID-19-associated stroke compared to non-COVID-19 associated [258]. In fact, to reduce the risk of mortality and progression of cryptogenic stroke, a study by the Society of Vascular and Interventional Neurology (SVIN) COVID-19 multinational registry had deduced a practical approach for clinicians in dealing with COVID19-associated small or large vessel stroke (i.e., with higher CRP, white blood cell count, and D-dimer levels) to consider that the presence of 5 risk criteria such as older age, male sex, diabetes, National Institutes of Health–Stroke Scale (NIHSS) 10 + , and cryptogenic stroke would imply an 80% chance of in-hospital mortality, with the risk of death with minimum of 3 criteria met [259]. Although this SVIN criteria warrant further external validation, it is useful in clinical management.

Additionally, the evidences on the use corticosteroids in the treatment for COVID-19 rest mainly on their high efficacy as an anti-inflammatory agent and therapy (i.e., in chronic inflammatory disease) [260]. Recent study also suggested the beneficial role of corticosteroids to reduce mortality in sepsis, alongside with the likely anti-inflammatory actions to reduce the detrimental effects of MPs-mediated inflammation [276]. Hence, corticosteroids may indirectly aid in reducing the peripherals MPs level which in turn could prevent undesirable progression and/or further adverse effect of the infection particularly towards the onset of CSVD. In this context, another study reported that COVID-19 patients with respiratory support administered with corticosteroid therapy (namely, dexamethasone, 6 mg per day for up to 10 days) had a reduced 28-day mortality [262]. Corroboratively, in September 2020, WHO published a guideline on the use corticosteroids for COVID-19 with a strong recommendation of systemic (i.e., intravenous, or oral) corticosteroid therapy (e.g., 6 mg of dexamethasone orally or intravenously daily or 50 mg of hydrocortisone intravenously every 8 h) for up to 10 days in patients with severe and critical COVID-19, as well as a conditional recommendation not to use corticosteroid therapy in patients with non-severe COVID-19 [263]. Nonetheless, a meta-analysis of over twenty thousand COVID-19 patients revealed a higher mortality among those who received corticosteroid therapy compared to those who did not (over 3 to 12 days treatment course). This may be due to the prothrombotic effect of the steroids which was doubled by their adverse drug reactions [264], in which the role of microthrombogenic MPs may be pertinent, albeit speculative. Thus, a rationalize use of corticosteroid is recommended to be guided by their risk–benefit ratio, whereby short course, i.e., up to 10 days therapy among a selected COVID-19 patient, may be beneficial, while an extended course may be detrimental [265].

Apart from hyperinflammation, cytokine storms mediated by hyper-coagulopathy and immunopathogenesis induced by SARS-CoV2 in vulnerable patients may lead to an increased mortality due to ARDS and MOD. Hence, the pragmatic approach such as decreasing the burden of aberrant coagulation, cytokine storm and viral loads through plasmapheresis therapy (with or without therapeutic plasma exchange [TPE]) had been reported to be beneficial in the management of COVID-19 [266]. Therapeutic plasmapheresis is the removal of abnormal accumulated substances (such as cytokines or autoantibodies) from the plasma [267]. In pre-clinical and/or clinical setting, the plasmapheresis can be carried out through plasma filtration (restricted to pores size of the filter-hence removal of molecules is limited) or centrifugation (unlimited removal of molecules) [268]. Besides, TPE (i.e., removal of toxins and pro-inflammatory cytokines that mediate CRS and ARDS) also has been suggested as a novel therapeutic approach for critically ill COVID-19 patients [269], whereby Zhang et al. reported that COVID-19 patients administered with TPE had a reduced 28-day of mortality and higher extubation rates [270]. In this regard, as discussed, higher concentrations of pro-inflammatory cytokines at the early stage of infection and inflammation may trigger the formation of MPs (with their detrimental roles) which in turn may lead to higher chances of future COVID-19 complications. Thus, an early consideration for therapeutic plasmapheresis (with or without TPE) may afford clinicians more effectively in disease prevention and/or progression.

At best, we have evidence to implicate multi-factorial prothrombotic states observed in COVID-19 which include heightened immuno-inflammatory responses (through mechanisms such as CRS, complement activation, endothelial injury), as well as possible contribution from systemic pressure dysregulation (RAS-related) [64, 248]. Some had suggested that SAR-CoV-2 itself can possibly activate the coagulation cascade through a mechanism that is yet to be uncovered [50, 248]. In this review, we highlight relevant evidence to relate SARS-CoV-2 infection with the risks of CSVD (both symptomatic and occult manifestations) through MPs-mediated micro- and macro-thrombosis, initiated in the peripheral (chiefly, the lungs) and potentially embolized to harm distally (i.e., the brain), or even as an in situ micro-thrombosis involving vulnerable end-arteries in cerebral microcirculation linked to CSVD.

## Conclusion

**Fig. 5:**
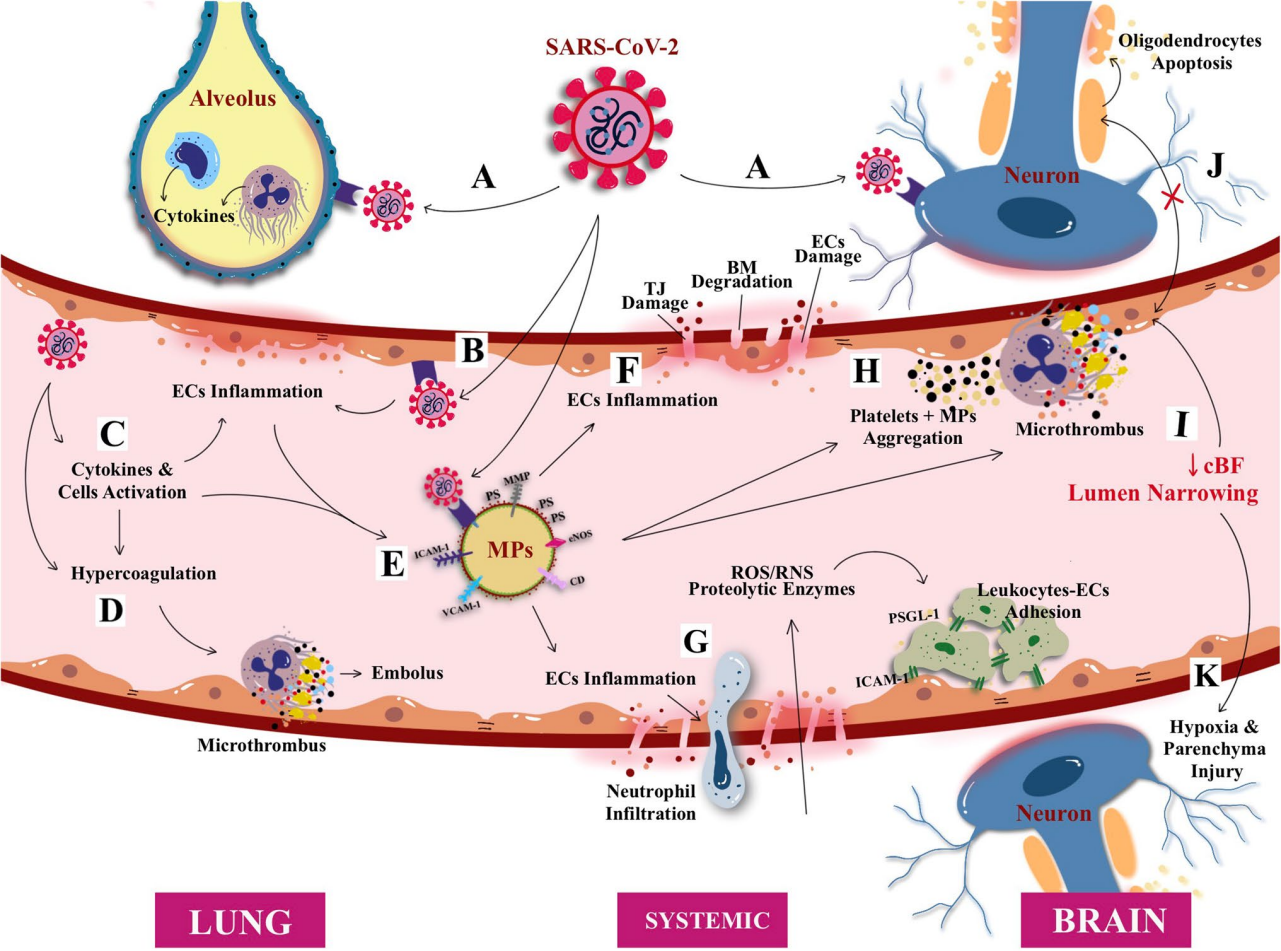
Proposed interaction between COVID-19 infection and the formation of circulating microparticles (MPs) as plausible microthrombogenic risk factor for cerebral small vessel disease (CSVD) in addition to existing co-morbidity with conventional CVD risk factors—for overt symptomatic stroke events to occult (asymptomatic) manifestations. **A** SARS-CoV-2 infection in lung alveolus and central nervous system through angiotensin converting enzymes type 2 (ACE2) receptor present on the surface of lung alveolus and nerves cells. **B** SARS-CoV-2 also enters vascular microcirculation causing endothelial cells (ECs) activation and inflammation, **C** cytokines releases causing further inflammation and cellular activation and **D** hypercoagulation causing elevated clots/microthrombus formation and embolus to other organ/s. **E** cytokines release and cellular activation induced the formation of circulating microparticles (MPs). **F** MPs bring surface matrix metalloproteinase that can cause ECs inflammation and induce blood brain barrier (BBB) disruption, through (1) tight junction (TJ) damage, (2) basement membrane (BM) degradation, and (3) the EC damage and dysfunction. **G** BBB damage and endothelial dysfunction elevate the cellular (i.e., neutrophil) infiltration and hence increase cellular oxidative stress through increment of reactive oxygen species (ROS), reactive nitrogen species (RNS), and proteolytic enzymes, followed by leukocytes-ECs adhesion on the endothelium lining hence causing arterial wall blockage. **H** MPs also cause aggregation and platelet aggregations at the endothelium wall causing lumen narrowing; besides, the thrombo-emboli from microcirculation also can settle at the wall and cause blockage and narrowing of lumen and reduce cerebral blood flow (CBF). **I** reduced CBF and lumen narrowing can cause **J** no crosstalk between ECs and neuronal oligodendrocytes and hence cause oligodendrocytes apoptosis, i.e., demyelination disease and **K** neuronal/glial hypoxia and cerebral parenchymal injury. Thus, this emerges as a potential pathogenesis of occult CSVD

MPs are pro-inflammatory, pro-coagulant membrane vesicles released by various cell types. In the setting of hemostatic imbalance such as that of COVID-19 infection, MPs are likely to be involved, even from the early sepsis as a mean to compensate for the host’s systemic inflammatory response. Importantly, MPs also may induce deleterious changes in the expression of enzyme systems related to inflammation and oxidative stress which are plausible during the different phases of COVID-19 infection. In fact, MPs (i.e., EDMPs) bearing ACE2 may systemically embolize from the site of formation (i.e., lung) to the distant target (i.e., brain), hence depositing the virus and further aggravate disease complications. Given the three phases of the COVID-19 infection, it is probable that the imbalance between coagulation, inflammation, and endotheliitis could progress from physiological body defenses (in early viremia phase) to pathological hyper-reaction (in pulmonary and hyperinflammation phases). Thus, it is plausible that the thrombosis initiated by the innate immune system that aims to limit SARS-CoV-2 dissemination ends with an anomalous functioning of this system that may have contributed to the endotheliitis, resulting in loss of thrombo-protective mechanisms, excess thrombin generation, fibrinolysis dysregulation, and thrombogenesis which attract various coagulation and inflammatory players as described in this review, with a particular emphasis on the role of MPs in CSVD pathomechanism, as illustrated in Fig. 5.

In conclusion, we hypothesize that MPs-mediated microthrombogenesis may play an important role in CSVD manifestations of COVID-19 patients through the course of the infective process. Research employing comprehensive panels of circulating MPs biomarkers for suspected and proven cases of COVID-19 may offer relevant clues to hypercoagulability and hyperinflammation states and extending this relationship to understand the manifestation of COVID-19-associated CSVD as we await the world to declare an affirmative win against this unprecedented twenty-first-century pandemic.

## Data Availability

Not applicable.
